# The Effect of Chromosomes on Courtship Behavior in Sibling Species of the *Drosophila virilis* Group

**DOI:** 10.3390/insects14070609

**Published:** 2023-07-05

**Authors:** Elena G. Belkina, Dmitry G. Seleznev, Svetlana Yu. Sorokina, Alex M. Kulikov, Oleg E. Lazebny

**Affiliations:** 1 Koltzov Institute of Developmental Biology, The Russian Academy of Sciences, 119334 Moscow, Russia; ellida69@mail.ru (E.G.B.); svetlana_ibr@mail.ru (S.Y.S.); amkulikov@gmail.com (A.M.K.); 2 Papanin Institute for Biology of Inland Waters, The Russian Academy of Sciences, 152742 Rybinsk, Russia; dmitriy@seleznev.name

**Keywords:** courtship behavior, *Drosophila virilis* group, video typing method, structural equation modeling, behavior genetics, sexual selection, X chromosome demasculinization

## Abstract

**Simple Summary:**

Courtship behavior has been the focus of research for a long time due to its association with one of the earliest and most effective mechanisms of interspecies isolation. The *Drosophila virilis* species group, which is a model for studying the mechanisms of evolution, has been studied extensively for various aspects of the courtship ritual, including the male courtship song. Despite genetic fine-mapping of this courtship behavior element, no genetic analysis of the ritual as a whole has been conducted. This study aimed to determine the effect of chromosomes on the latency and duration of courtship-ritual elements in the *D. americana* and *D. virilis* species system. Since the expression of the courtship-ritual structure depends on the behavior of both the female and the male, to study the inheritance of the courtship elements’ parameters, tests were carried out with reference partners (females and males of the original species) of representatives of different generations: parental, F_1_, F_2_, and backcrosses (F_B_). Structural equation modeling was used to process the parameter data for a series of courtship-ritual elements. The obtained results indicated that species-specific variability in courtship-element parameters in females is concentrated on the autosomes, while in males, it is concentrated on the X chromosome. This influence of the X chromosome in successful male courtship behavior may be a contributing factor in the lack of complete X chromosome demasculinization observed in Drosophila.

**Abstract:**

Prezygotic isolation mechanisms, particularly courtship behavior, play a significant role in the formation of reproductive barriers. The action of these mechanisms leads to the coexistence of numerous closely related insect species with specific adaptations in a shared or adjacent territory. The genetic basis of these mechanisms has been studied using closely related Drosophila species, such as the *D. virilis* group. However, the investigation of individual courtship behavior elements has been limited until recently, and the effect of genotype on the species-specific features of courtship as a whole has not been thoroughly examined. It should be noted that courtship behavior is not a typical quantitative trait that can be easily measured or quantified in both females and males, similar to traits like wing length or bristle number. Each courtship element involves the participation of both female and male partners, making the genetic analysis of this behavior complex. As a result, the traditional approach of genetic analysis for quantitative traits, which involves variance decomposition in a set of crosses, including parental species, F1 and F2 hybrids, and backcrosses of F1 to parental species, is not suitable for analyzing courtship behavior. To address this, we employed a modified design by introducing what we refer to as ‘reference partners’ during the testing of hybrid individuals from F1, F2, and backcrosses. These reference partners represented one of the parental species. This approach allowed us to categorize all possible test combinations into four groups based on the reference partner’s sex (female or male) and their constant genotype towards one of the parental species (*D. virilis* or *D. americana*). The genotype of the second partner in the within-group test combinations varied from completely conspecific to completely heterospecific, based on the parental chromosomal sets. To assess the contribution of partner genotypes to the variability of courtship-element parameters, we employed structural equation modeling (SEM) instead of the traditional analysis of variance (ANOVA). SEM enabled us to estimate the regression of the proportion of chromosomes of a specific species type on the value of each courtship-element parameter in partners with varying genotypes across different test combinations. The aim of the current study was to analyze the involvement of sex chromosomes and autosomes in the formation of courtship structure in *D. virilis* and *D. americana*. The genetic analysis was complemented by video recording and formalization of courtship-ritual elements. *D. virilis* was found to be more sensitive to mate stimuli compared to *D. americana*. The majority of species-specific parameters, such as latency and duration of courtship elements (e.g., male and female song, following, licking, and circling), were shown to be influenced by the *D. virilis* genotype. However, not all of these parameters significantly impact copulation success, with the male song, licking, and following being the most significant. In females, the female song was found to have a significant relationship only with copulation duration. The influence of the female genotype on the species-specific parameters of courtship elements is primarily related to autosomes, while the male genotype is associated with the X chromosomes. The study suggests that sexual selection primarily occurs through acoustic and chemoreceptor channels.

## 1. Introduction

Courtship behavior is the most widespread form of innate behavior in the animal kingdom. Courtship rituals can vary greatly even among closely related species and aim to address two issues: first, to identify a conspecific partner to avoid gene-pool erosion and prevent unproductive interspecific crossings, and second, during the ritual, for the female to assess the potential partner’s ability to produce a healthy and fit offspring. Consequently, the success of courtship relies on both the male’s activity and perseverance, as well as the female’s choice. The female possesses a repertoire of signals, including positive stimuli such as wing spreading and genital plate expansion, and negative stimuli such as avoidance, repulsion, and the release of antiaphrodisiacs. At any given moment, the female has the ability to deliver a negative or positive signal, thus interrupting or progressing the courtship program to its final phase [1]. Therefore, courtship behavior is linked to the reproductive isolation of sibling species, and understanding the genetic basis of courtship behavior is important for studying the genetic mechanisms of speciation.

The Drosophilidae family is an excellent model system for studying the genetic basis of courtship behavior and reproductive isolation. *Drosophila melanogaster* is the most extensively studied species in this regard and is genetically the most well-understood species. However, there exist groups of partially isolated sibling species in Drosophila that can produce fertile offspring, which can be utilized for generating F_2_ and backcrossing with parental species in laboratory settings for genetic analysis purposes.

Courtship behavior in *Drosophila* is typically multimodal, involving the use of multiple sensory channels to exchange several sensory signals sent and received by both sexual partners during courtship. Courtship communication occurs through chemical, acoustic, tactile, and visual stimuli that are species-specific and aid in the recognition of mating partners. *Drosophila* males’ courtship behavior typically consists of a sequential series of stereotypical elements, including turning towards the female, tapping her abdomen with their foreleg tarsi, wing vibration, circling the female, and licking her genitalia. During tapping and licking, both sexes receive chemical and tactile cues from each other, while male wing vibration produces acoustic and sometimes visual cues (such as in [1,2,3,4,5,6]). Drosophila females respond differently to male courtship depending on the species. For instance, *D. virilis* females may vibrate their wings to produce songs during courtship, with both sound and other modalities being used to coordinate acoustic duets between mating partners [7].

Despite the complexity and multilevel nature of courtship behavior, the spatial and temporal relationships of courtship-ritual elements can be relatively easy to quantify. Various authors have attempted to quantify the elements of courtship behavior and represent their sequence graphically using ethograms or kinetographs [8,9,10,11,12,13,14,15]. However, for the *D. virilis* group, the use of arrow diagrams is not suitable because some elements of the courtship ritual occur simultaneously instead of sequentially. Of particular significance is the study conducted by Nickel and Civetta [16], who assessed the magnitude of the effects of chromosomes on the courtship-ritual structure in three sibling species of the *D. virilis* group. The authors evaluated the courtship behavior of conspecific and heterospecific pairs through male activity, which was measured by a cumulative male courtship progressive score. One disadvantage of this method was the inability to take into account the effects of individual elements.

Unlike other quantitative traits, courtship behavior in *Drosophila* is a joint product of both female and male individuals. Each courtship element exhibited by both partners is contingent upon their interaction during the courtship ritual. As a result, the parameters of the analyzed elements are influenced by the genotypes of both partners. The conventional method of genetic analysis for quantitative traits using variance decomposition (ANOVA) does not account for the effects of the genomes of females and males interacting during the courtship ritual. Our approach involves a consistent effect from a partner with a reference genotype and a variable effect from partners with changing genotypes. We conducted four groups of behavioral experiments with thirty individual pairs of courtship mates tested in each. In these experiments, we studied changes in the duration and latency of courtship-ritual elements. The genotype of one courtship mate was constant and represented a parental genome (*D. virilis* female, *D. americana* female, *D. virilis* male, or *D. americana* male), while the genotype of the opposite-sex partner gradually changed from conspecific to heterospecific through a series of hybrid genotypes.

The most appropriate method for analyzing the proposed scheme is the structural equation modeling method. Structural equation modeling is a set of multivariate techniques widely employed in social and behavioral research fields [17,18,19]. We follow the classical genetics assumption that the cumulative effect of genetic factors linked to a particular chromosome is expressed in the effect of this chromosome on certain traits [20]. This study examines the overall effect of the autosomes and X chromosomes of different species on the parameters of courtship elements and copulation.

Therefore, the primary objectives of this study were to assess the contribution of sex chromosomes and autosomes in males and females to the species-specific elements of the courtship ritual and to propose a model for analyzing traits that are influenced by the interaction between two partners.

## 2. Materials and Methods

### 2.1. Drosophila Stocks and Crosses

All sibling species used were obtained from the collection of the Koltzov Institute of Developmental Biology: *D. virilis* (strain 102, Berlin, Germany, 1967) and *D. americana* (strain 403, Texas Univ., Prof. Stone, 1965, Austin, TX, USA).

We conducted interspecific hybridization where ♀♀ *D. americana* were crossed with ♂♂ *D. virilis* to obtain hybrid progeny F_1_, F_2_, and backcross progeny (F_B_) (Figure S1). However, we were unable to obtain offspring from the reciprocal variant of crossing ♀♀ *D. virilis* × ♂♂ *D. americana* due to the presence of postmating prezygotic isolation, as documented in prior literature [21,22]. The flies were reared on a semolina–yeast medium in glass vials (100 × 25 mm) at 21 ± 0.5 °C under a standard 12-h light/12-h dark cycle. One-day-old flies were immobilized using cold anesthesia and separated by sex.

### 2.2. Behavior Tests

**Figure 1:**
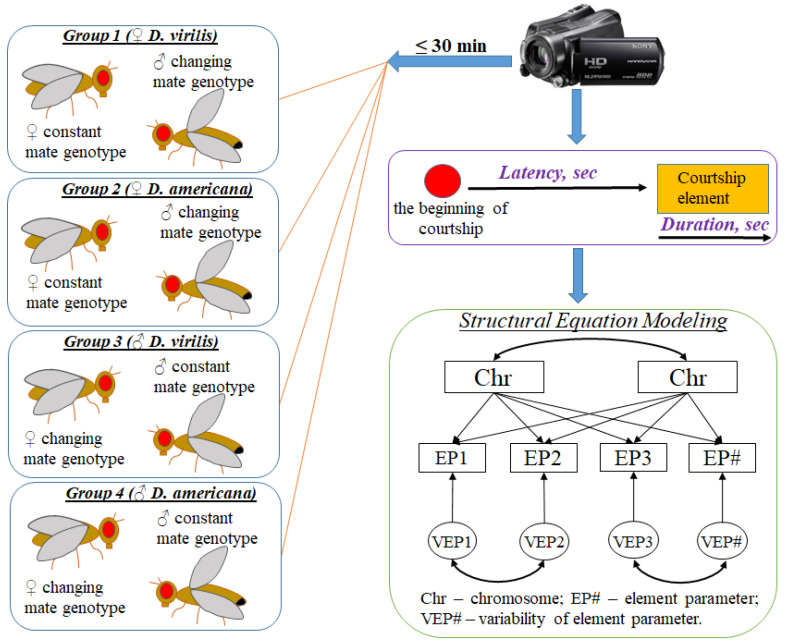
Experimental Design. Experiments were grouped based on a specific rule: one partner with a consistent parental genotype, referred to as the partner with a constant genotype and another partner with a genotype that varied from conspecific to heterospecific across a series of experiments. The courtship ritual was captured using a video camera and subsequent video analysis allowed for the identification and separation of individual courtship elements. The genetic analysis of courtship behavior traits involved the integration of various methods, including structural equation modeling (SEM). MA more detailed representation of the genetic part of this experimental design is provided in Figure S1.

We conducted no-choice behavioral tests to identify the mating behavior characteristics necessary for successful ritual and copulation. A total of twenty experiment variants were performed in this study (Table 1). Each experiment involved thirty individual pairs of Drosophila females and males, and the experimental design is delineated in Figure 1. To evaluate the impact of genotype on each courtship element from both partners, we employed the following experimental design. The nonchoice tests were divided into four groups based on the sex and species specificity of the reference partner and the changing genotype of the opposite partner. Each group consisted of six experimental variants, which were ranked according to a gradual change in the genotype of the opposite partner from one conspecific to heterospecific. Each experimental variant consisted of 30 individual tests (pairs) with the mandatory involvement of a reference partner, while the opposite partner represented the genotype of the same parental species (conspecific test), or the genotype of a different parental species (heterospecific test), or a hybrid genotype from F_1_, F_2_, or F_B_ generations (partially heterospecific test) (Table 1). The proportion of sex chromosomes and autosomes of *D. virilis* and *D. americana* in the opposite partners or partners with a changing genotype (represented by F_2_ and F_B_) was used to determine the influence of genotype on quantitative traits [23], specifically the parameters of the courtship elements. This approach enabled us to consider the medians and quartiles of the traits. Within each group of experiments, we assessed the effects of a partner with a changing genotype on the variability of courtship element parameters and the efficiency of copulation.

Virgin females and males were kept separately in vials and used in the mating tests only upon reaching sexual maturity. Hybrid males resulting from interspecies crosses often exhibit delayed maturation. At 25 °C, 49% of hybrid males aged 5–7 days from the cross between *D. virilis* and *D. lummei* were observed to be sterile, while only 4–7% of males were sterile after 14 days [24,25,26]. We conducted experiments that demonstrated that up to 90% of hybrid males resulting from the cross between *D. virilis* and *D. americana* became fertile and began actively courting females after an extended maturation period. The maturation period for *D. virilis* was reached at 14 days, for *D. americana* at 21 days, for F_1_ offspring at 42 days, for F_2_ at 28 days, and for F_B_ at 21 days. Each fly was used in only one test. Within each experimental group, one partner’s genotype remained constant while the other partner’s genotype gradually changed from conspecific to completely heterospecific (Table 1; Figure S1 and Figure 1).

To record courtship behavior, we utilized a video recording method where all interactions between one female and one male were captured using a Sony HDR-SR 12E video camera (Japan) and subsequently analyzed using Virtual Dub 1.10.3 software. The experimental protocol was initiated by placing one female and one male into a glass vial (100 × 25 mm) with 7–8 mL of the standard medium by gentle aspiration without anesthesia. If the male exhibited no interest toward the female within 30 min of the experiment’s commencement, the partners were separated. In the case of a successful courtship, the behavior was recorded until the flies copulated or until 30 min had elapsed. Recording commenced upon the male displaying interest in the female, usually beginning with tapping. The latencies from the experiment’s start to the initiation of each courtship element and the durations of each courtship element were recorded for each pair (Figure 1). If an element was interrupted and resumed, its total duration was represented by the sum of individual periods’ durations. If an element was entirely absent in the ritual evaluated for a given pair, the element received the maximum latency, exceeding the total evaluation time by one second (1801 s = 30 min plus one second), and a duration of 0 s. Eight courtship elements were identified, including following (the male follows the female), tapping (the male touches the female abdomen by the forelegs), licking (the male licks the end of the female abdomen), male singing (the male takes one wing aside and vibrates by it), female singing (the female vibrates by both wings being almost folded), circling (the male circles around the female), copulation attempt (the male tries to mount the female but the attempt lasts for less than one minute), and copulation [27,28,29].

### 2.3. Genetic Analysis of Courtship Traits

Genetic analysis of courtship behaviors was conducted using a set of methods collectively referred to as structural equation modeling (SEM). SEM involves the creation of a structural model to assess the effect of chromosomes on courtship-element parameters and, indirectly, copulation parameters. The goal of this analysis was to determine how increasing differences in genotype between partners impacted courtship behaviors and copulation parameters when one partner’s genotype remained constant.

We utilized the Amos software module [30] within the IBM SPSS statistical software package (IBM Corp. Released 2015. IBM SPSS Statistics for Windows, Version 23.0. Armonk, NY, USA: IBM Corp.) to conduct our analysis.

The initial data analysis involved constructing structural models under the following conditions: (1) the Y chromosome indirectly impacts the courtship-ritual elements by interacting with the X chromosome and autosomes; (2) the X chromosomes and autosomes have an optional direct effect on courtship-ritual elements in both partners; (3) the chromosomes have an indirect effect on copulation-efficiency traits, such as latency and duration, through their direct effect on courtship elements; (4) traits such as “following” and “copulation attempts” do not exhibit significant differences in most experiments and were, therefore, excluded from the model. To account for the original data distribution’s skewness and reduce scaling effects, we transformed all data using a base-10 logarithmic function.

**Figure 2:**
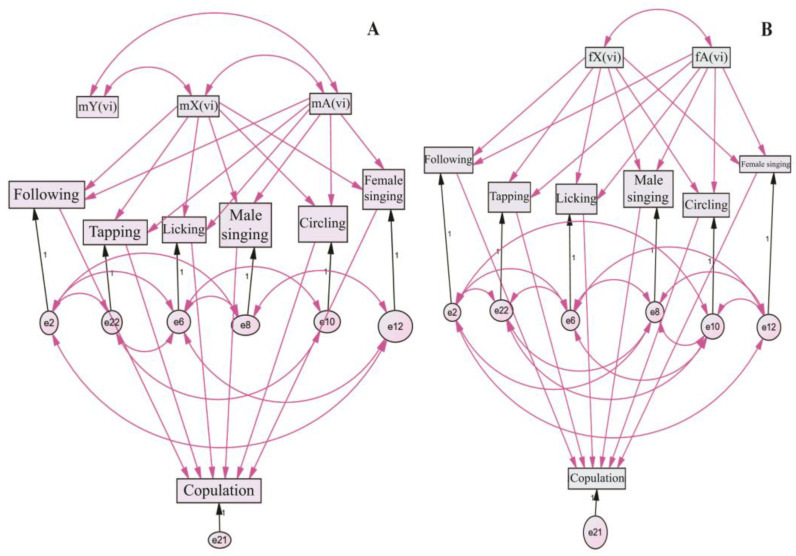
Genetic variability models of courtship-ritual element latency traits in pooled pairs with a partner of constant genotype: (**A**) *D. virilis* females and (**B**) *D. virilis* males. Key: m—male; f—female; X, Y—sex chromosomes; A—autosomes; vi—*D. virilis*. Double-sided arrows represent correlations and potential mutual regulatory interactions between the elements. One-sided arrows indicate the regulatory influence of higher elements on lower ones. Violet arrows denote optional connections, while black arrows indicate required connections. Rectangles represent external and internal variables, while circles denote the variability of dependent or internal variables. The symbols e2–e22 represent sets of variables that characterize the variability of internal variables.

In the second stage, we conducted an exploratory analysis that involved selecting one or several models from a large set of candidate models that best corresponded to the observed variability. We used either a stepwise or fully-fledged heuristic search to find the best models. The set of candidate models was determined by the constant composition of structural elements (i.e., genetic and courtship elements) and the links between them. All links were considered optional, meaning the algorithm had to evaluate models with a complete absence of connections to a complete set. Structural models were designed by taking into account regulatory relationships between chromosomes (i.e., genetic factors located on chromosomes), relationships between chromosomes and courtship elements, and correlations between courtship elements and their effect on copulation parameters. Figure 2 shows examples of basic models for the influence of genetic factors linked to the X chromosome and autosomes on the latency of courtship elements and copulation for a group of experiments involving males (Figure 2A) and females (Figure 2B) with a constant genotype. We also present the dependence of courtship-element latency on the partner’s genotype, which was not constant.

To eliminate trait collinearity in structural models, we used only one set of chromosome (*D. virilis*) frequencies. We combined any two traits where the correlation between them was equal to, or greater than, 0.95, formally leaving only one trait.

Confirmatory analysis was used to study the obtained models and estimate the effects of *D. virilis* chromosomes on courtship elements and on copulation efficiency as the final stage of courtship behavior. None of the analyzed traits’ distributions corresponded to the multivariate normal distribution, and the total sample size was approximately five times larger than the number of analyzed model parameters. Therefore, nonparametric methods were used for the analysis, including unweighted least squares (ULS) and scaleless least squares (SLS). The asymptotically distribution-free estimation (ADfE) method, which has a significant set of determinable statistical criteria, was also used. However, ADfE requires very large sample sizes for accurate estimates [31]. Due to insufficiently large sample sizes, the standard error of the mean exceeded the value of the mean. The most appropriate analysis method was selected using evaluation criteria for model goodness of fit to empirical data: root mean square residual (RMR), goodness-of-fit statistic (GFI), adjusted goodness-of-fit statistic (AGFI), parsimony goodness-of-fit index (PGFI), and methods based on the proportion of bootstrap samples that did not fit the model when bootstrap estimates were available. The sample size for each analyzed model was lower than recommended for the given number of estimated parameters, increasing the probability of erroneous estimates and limiting method usage. For the *D. americana* female group, a bootstrap of SLS-SEM and ULS-SEM regression coefficients was available to estimate the coefficients’ variability and assess the direct and indirect effects. When analyzing the latency of courtship elements for experimental Group 1 (*D. virilis* female), only a Bollen–Stine bootstrap was available, determining model accuracy.

When estimating the effect of *D. virilis* chromosomes on courtship elements in each experimental group, we used standardized regression coefficients of the proportion of *D. virilis* chromosomes on courtship-element parameters as obtained from solving the corresponding models. Standardizing the regression coefficients allowed us to compare their values obtained from different models.

## 3. Results

### 3.1. Variability of Courtship Elements in Courtship-Partner Pairs with Various Genotype Combinations

Table 2 shows the results of our analysis of courtship-element variability in the pooled sample consisting of all experimental groups of courtship partner pairs.

The additional results in Tables S1–S20 provide detailed information on the individual variability of these elements in each of the 20 experiments. Minimum values and lower quartile values equal to or close to zero in the latency values of a certain element mean the implementation of this element by a certain number of partners from the beginning of the courtship. A zero-median latency value of an element suggests the initiation of this element from the start of the courtship in a significant number of pairs. As per the observations, most pairs typically commence the courtship ritual with tapping. Conversely, a duration value of 0 and a maximum latency value of 1801 s indicate that this element was not executed during the observation period. According to the data presented, this applies to elements such as following, circling, and copulation attempts. The absence of an element could result from an early termination of the courtship ritual.

Significant differences in the mean and median values suggest a deviation from the normal distribution of values for nearly all courtship elements. This assertion is corroborated by the high kurtosis and skewness coefficients recorded in most courtship elements. However, an examination of the data presented in Tables S1–S20 demonstrates substantial differences in the median values and diversity of courtship traits between distinct experiments. To verify these dissimilarities, courtship-trait values were juxtaposed across the experiments in each group.

As heterospecificity between partners increased, at least half of the courtship traits exhibited significant changes in their values (Figure S2). Thus, tapping duration increases as the heterospecificity of males increases. Licking duration also increases with a higher proportion of the *D. americana* genotype in males and decreases with a higher proportion of the *D. virilis* genotype. Both elements do not show a significant dependence on the genotype of females in tests with reference males. However, licking latency demonstrates a dependence on the *D. virilis* genotype in both males and females, reaching maximum values, i.e., termination of the ritual, in fully heterospecific pairs. The duration and latency of singing in males and females exhibit dependence on the chromosomal composition in both sexes. In this case, female singing is more influenced by the composition of female chromosomes, while male singing depends on the composition of male chromosomes. The increase in heterospecificity affects the duration of circling, and the expression of this trait depends on the sex of the partner with a constant genotype, suggesting the involvement of sex chromosomes in the manifestation of this element. A detailed analysis of the variability of courtship-ritual elements is provided in the Supplementary Materials (Text S1, Figure S2, Tables S1–S24).

The copulation frequency in heterospecific pairs with the ♀ *D. americana* + ♂ *D. virilis* combination (Groups 2 and 3) is extremely low during the 30-min observation period. Only one instance of copulation was observed shortly after the initiation of courtship (10 s) and it lasted for one and a half minutes. In the reciprocal mating test (Groups 1 and 4), copulation occurred in 37% of cases, with the maximum duration exceeding three minutes. Notably, when at least 50% of the chromosomes from the second species were present, the ability to copulate was nearly completely restored.

The success of the courtship ritual is effectively represented by the proportion of copulating pairs in each experiment (Table 1). In all four experimental groups, the significance of observed differences in the proportion of successful and unsuccessful copulations within the tested pairs was estimated by using ratios from conspecific tests as a control. In Group 1, significant differences were observed for completely heterospecific pairs and pairs with males from F_1_ and F_2_. In Groups 2 and 3, a significant difference in copulation efficiency was noted only in completely heterospecific tests. Lastly, in Group 4, significant differences in copulation efficiency were observed for pairs with hybrid females and completely heterospecific pairs.

Thus, we observe an “asymmetric” pattern in the proportion of copulating pairs within an experimental group, which is significantly different from the control, both in groups with the same sex of partners with a constant genotype (Groups 1–2 or 3–4) and in groups with the same species of males and females with a constant genotype (Groups 1–3 or 2–4). This pattern does not allow us to draw a definitive conclusion regarding the predominant contribution of sex chromosomes to the effectiveness of the courtship ritual but suggests their involvement at the very least. To assess the effect of sex chromosomes and autosomes on the components of the courtship ritual, a structural equation modeling approach was employed in the analysis of the four groups.

### 3.2. Selection of Optimal Candidate Models

The process of model specification involved enumerating all possible derivative versions of the base model, ranging from the absence of any links between its elements to a complete set of links, including two-way correlations and one-way regulatory connections. During the analysis, information criteria were used to assess the agreement between the variability predicted by the models and the observed variability (Table 3). For all four experimental groups and each set of data on courtship latency and duration, 14 optimal models were selected. The minimum values of the information criteria range from one to four within each optimal model. Models that possess minimum information criteria values but lack associations between genetic elements linked with X chromosomes and autosomes, as well as courtship traits, are excluded from the table.

The base models examining the relationship between genetic variability and the variability of courtship elements in pairs involving females with a constant genotype exhibited minor differences in the latencies and durations of courtship elements. The models for latency values encompassed 36 estimated parameters, including three external variables represented by male sex chromosomes and autosomes, as well as six internal variables represented by courtship elements.

The direct effect of the Y-chromosome on the parameters of the courtship elements was not considered for several reasons.

1. The Y chromosome carries a very small number of protein-coding genes, just over two dozen, which is significantly fewer than the set of genes present on the X chromosome and autosomes;
2. In the case of these two species, only one direction of crosses is possible to obtain F_1_, namely a female *D. americana* and a male *D. virilis*. Consequently, it is not possible to obtain a complete combination of chromosomes from both species, including those with the Y-chromosome of *D. americana*. Offspring with the corresponding Y chromosome were only obtained from crossing F_1_ females with *D.* americana males. It is evident that *D. americana* males also possessed the Y chromosome of their species. The analysis of the role of sex chromosomes and autosomes included six male genotype combinations, with a single *D. americana* Y chromosome genotype in Group 1 and two genotypes in Group 2 (Table 1);
3. Comparisons of test groups differing in the species origin of the Y chromosome are possible between the male genotypes Y^Am^/X^(Am,Vi)^; A^(Am,Vi)^/A^Am^, and Y^Vi^/X^(Am,Vi)^; A^Am^/A^Vi^, which have an equal ratio of X chromosomes of both species and a 25% advantage of *D. virilis* autosomes in the second case, when evaluating their traits in pairs with *D. americana* females. For all traits, comparisons using the Mann–Whitney U Test and Wald–Wolfowitz Runs Test did not reveal significant differences (Tables S25 and S26).

Within the model, genetic elements associated with the Y chromosome did not have an independent connection with the courtship elements but instead displayed correlation connections with the X chromosome and autosomes, simulating regulatory connections. Genetic regulations within the model indirectly influenced copulation latency through other courtship elements. To address collinearity in the model, the variability of courtship elements in terms of durations was used, resulting in a formal combination of tapping and licking elements. This adjustment led to a reduction in the number of estimated parameters to 34 and a decrease in the number of internal variables to five. Similarly, the base models constructed for pairs involving males with a constant genotype followed a similar structure, with the distinction of the absence of the Y chromosome and its interactions. Typically, the identified optimal models exhibited a reduced number of connections, both in terms of regulatory (one-way) and correlation (two-way) relationships.

The subsequent stage of the analysis involved selecting a suitable method for solving the identified optimal models. Nonparametric methods were employed, aligning with the nature of the analyzed variability. Based on the information criteria values (Table S27), the unweighted least squares (ULS) method was found to be the most appropriate for model analysis in the majority of cases. However, the model-fit estimates obtained using the scale-free least squares (SLS) method yielded similar results in most instances. When analyzing the models constructed for these experimental groups, the results were compared using both methods, provided that bootstrap estimates of the variability of the standardized regression coefficients were obtained in both cases.

### 3.3. Indirect Influence of Chromosomes on Copulation Success

Table 4 illustrates the influence of chromosomes on copulation through the collective action of all considered courtship elements. In each of the four experimental variations, the status of X chromosomes and autosomes, which exhibit species specificity in *D. virilis*, undergoes changes in only one partner, while the genotype remains constant in the other partner. For ease of reference, we will refer to the X chromosomes and autosomes as X*^D. virilis^* and Aut*^D. virilis^*, respectively. The change in status corresponds to a variation in the proportion of the corresponding chromosomes in the given partner, ranging from zero to one. The standardized regression weight of the *D. virilis* chromosome proportion on a trait value quantifies the impact of the chromosomes on a specific trait. This effect is influenced by both changes in the trait value and the chromosome composition. It is anticipated that the effect of chromosomes will exhibit significant variations, potentially even reversing the original effect, when experiments involve partners (either males or females) with constant genotypes but from different species.

The impact of X*^D. virilis^* in males, when paired with a female of constant *D. virilis* genotype, manifests as a reduction in copulation latency (indicated by a negative regression coefficient), which aligns with expectations as copulation tends to occur earlier in conspecific tests. Particularly intriguing is the finding that the influence of X*^D. virilis^* in males is considerably more substantial than the effect of autosomes, which appears to be close to zero. As anticipated, the introduction of *D. americana* females yields the opposite outcome: the effect of X*^D. virilis^* in males leads to an elongation of latency. This can be attributed to the shift in heterospecificity observed when transitioning from X*^D. americana^* to X*^D. virilis^* in males. Furthermore, the effect of autosomes exhibits an opposing sign and is of a smaller magnitude compared to the effect of the X chromosome.

In pairs with a constant female genotype, the male genotype has an opposite effect on copulation duration, which is understandable considering that copulation duration in Drosophila is linked to insemination completeness and decreases during heterospecific tests. When paired with a *D. virilis* female, the X*^D. virilis^* genotype in males does not significantly impact copulation duration, whereas the Aut*^D. virilis^* genotype positively affects and increases copulation duration. However, the scenario changes when paired with a *D. americana* female; X*^D. virilis^* has a pronounced negative effect, while the effect of Aut*^D. virilis^* is nearly negligible. In this case, the decrease in copulation duration is primarily attributed to the male’s X chromosome.

In pairs with a constant male genotype, the significance of X chromosomes is greatly diminished in most cases. The presence of X*^D. virilis^* in females increases copulation latency, although the effect size is smaller compared to Aut*^D. virilis^*, which actually reduces copulation latency. The combined impact of genetic factors in females manifests as an extended copulation latency when tested with a *D. virilis* male, particularly with an increase in partner heterospecificity. In the same way, modifying the male genotype in a similar experiment yields a comparable effect (Table 4, latency, ♂ *D. americana*); the influence of X*^D. virilis^* is nearly neutral or negative, while the influence of Aut*^D. virilis^* is positive and substantially larger. Consequently, the overall effect is positive, resulting in a prolonged latency with an increase in heterospecificity.

In pairs with a constant *D. virilis* male genotype, the copulation duration is influenced by the female genotype, with X*^D. virilis^* exerting a negative effect and Aut*^D. virilis^* displaying a positive effect on this trait. The impact of X chromosomes is significantly smaller, approximately two or more times inferior to the effect of autosomes, and exhibits an opposite direction. As anticipated, the cumulative effect of genetic factors in this experimental setup will be positive, resulting in an increased copulation duration with reduced heterospecificity. Surprisingly, altering the male genotype to *D. americana* does not affect the effect of X*^D. virilis^* on this trait but drastically reduces the impact of autosomes to significantly negative values. Consequently, the overall effect of the genotype will be negative, leading to a decreased copulation duration with an increase in the heterospecificity of pairs.

The results lead to the conclusion that sex chromosomes and autosomes exert opposite effects on the copulation parameters. The influence of genetic factors, which are differentially expressed in males and directly impact courtship elements while indirectly affecting copulation traits, is predominantly determined by the X chromosome. Conversely, differentially expressed genes in females are primarily associated with autosomes, except for Group 4, where the effect of X chromosomes on copulation duration is significantly more pronounced compared to the effect of autosomes. This effect is further observed when *D. americana* males court females with a different proportion of *D. virilis* chromosomes.

### 3.4. The Effect of Chromosomes on Courtship Elements

**Figure 3:**
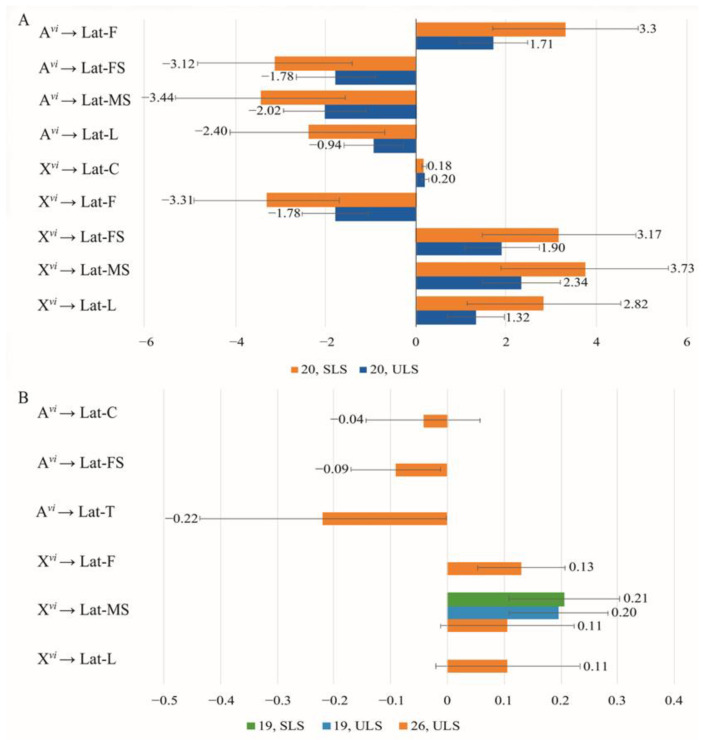
Effect of the proportion of *D. virilis* chromosomes in one courtship partner on the latency of courtship elements with the other partner having a constant *D. americana* genotype: (**A**) Genotypes change in males while females have a *D. americana* genotype (Group 2); (**B**) Genotypes change in females while males have a constant *D. americana* genotype (Group 4).

Both species-specific characteristics of a phenotype and courtship elements are dependent on the proportion of chromosomes from each species present in the genomes of the mating partners. In experimental Groups 2 and 4, *D. americana* served as the partner with a constant genotype. Notably, these groups exhibited a significant reduction in the standardized regression coefficients’ values for the proportion of chromosomes in the latency of courtship elements in females (Figure 3B) compared to males (Figure 3A). Additionally, the majority of latency values were low and did not significantly differ from zero, except for the impact of female X chromosomes on male following and singing. The effect of autosomes on latency is consistent between males and females only for the female singing element (Figure 3A,B), resulting in decreased latency for this element with an increase in partner heterospecificity. Conversely, the effect of X chromosomes on latency in males and females is similar for male singing and licking, leading to increased latency for these elements with increased heterospecificity.

When one partner in a pair has a constant *D. americana* genotype, there is a broader range of courtship elements influenced by the male’s genotype compared to those influenced by the female’s genotype. Notably, the effects of autosomes and X chromosomes in males on the latency of courtship elements exhibit opposite directions.

**Figure 4:**
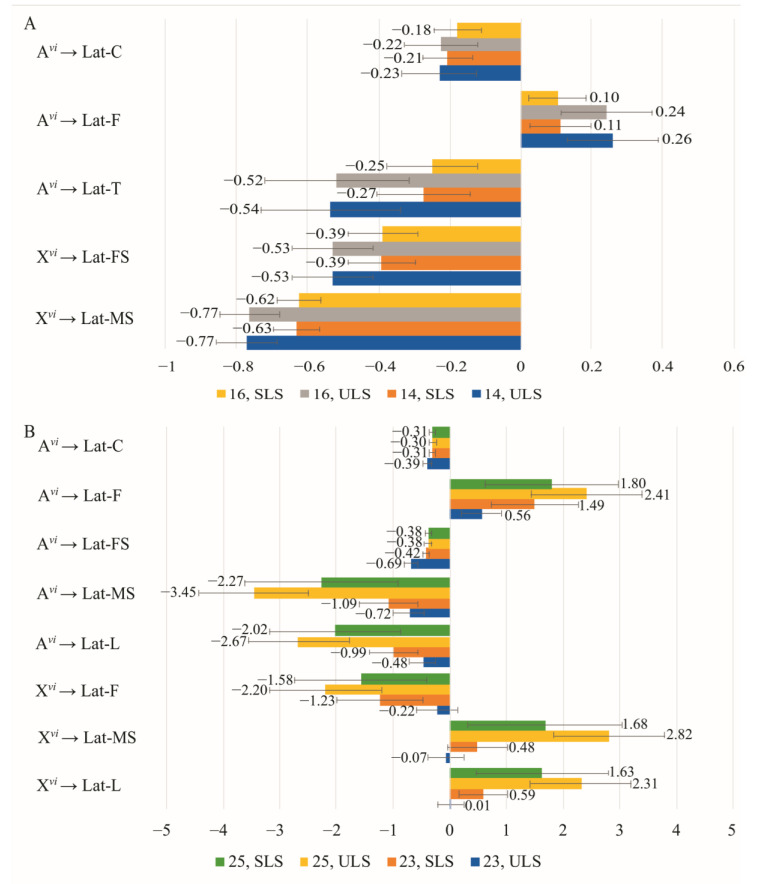
Effect of the proportion of *D. virilis* chromosomes in one courtship partner on the latency of courtship elements with the other partner having a constant *D. virilis* genotype: (**A**) Genotypes change in males while females have a *D. virilis* genotype (Group 1); (**B**) Genotypes change in females while males have a constant *D. virilis* genotype (Group 3).

When *D. virilis* is used as a partner with a constant genotype, a change in chromosome composition results in an elevation of regression coefficient values for the proportion of chromosomes on latency values of courtship elements in females. Interestingly, these regression coefficients even surpass those observed in males (Figure 4A,B).

The effect of autosomes on circling and following latencies is comparable in both males and females (Figure 4A,B). Increasing autosomal heterospecificity results in an elevation of circling latency and a reduction in following latency. Notably, the *D. virilis* X chromosomes have opposite effects on male singing latency in males and females, while also significantly impacting female singing latency. In pairs with *D. virilis* females, an increase in male heterospecificity leads to an extended male singing latency. Conversely, an increase in female heterospecificity in pairs with *D. virilis* males causes a decrease in female singing latency.

In pairs where one partner has a constant *D. virilis* genotype, the repertoire of courtship elements in females differs from and is broader compared to that in males. The effects of autosomes and X chromosomes in females on the latency of following, male singing, and licking exhibit opposite directions, with autosomes exerting a larger negative effect on these elements compared to the positive effect of X chromosomes.

**Figure 5:**
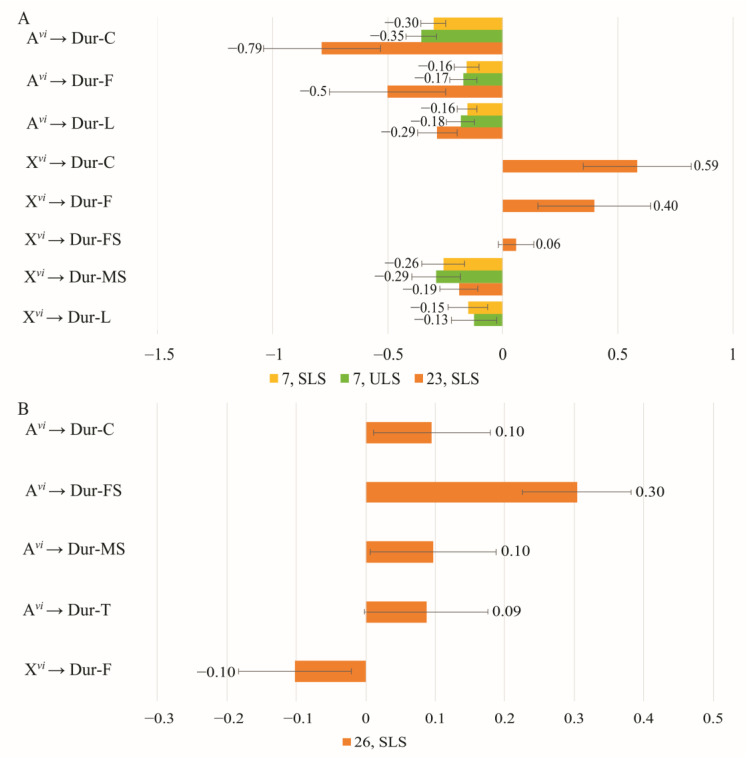
Effect of the proportion of *D. virilis* chromosomes in one courtship partner on the duration of courtship elements with the other partner having a constant *D. americana* genotype: (**A**) Genotypes change in males while females have a *D. americana* genotype (Group 2); (**B**) Genotypes change in females while males have a constant *D. americana* genotype (Group 4).

In pairs where one partner has a constant *D. americana* genotype, a change in chromosome composition leads to an increase in the standardized regression coefficient values of the proportion of *D. virilis* chromosomes on the duration of courtship elements in males, compared to females (Figure 5A,B). The sets of courtship elements for males and females exhibit significant differences, except for circling, which is influenced by *D. virilis* autosomes and displays regression coefficients of opposite signs in males and females. Consequently, this divergence in regression coefficient signs corresponds to a decrease in circling duration with an increase in heterospecificity in males and an increase in circling duration with an increase in heterospecificity in females.

The repertoire of courtship elements influenced by male autosomes includes circling, following, and licking, with their durations decreasing as autosomal heterospecificity increases (Figure 5A). Female autosomes significantly impact female singing, resulting in increased duration with higher levels of autosome heterospecificity (Figure 5B). The presence of the *D. virilis* X chromosome in males paired with *D. americana* females leads to extended circling and following durations, while male singing and licking durations decrease. In females, X chromosomes have a minor effect on following, reducing its duration with increased X chromosome heterospecificity. The effects of male autosomes and X chromosomes on circling and following durations exhibit opposite directions, with the effect of autosomes slightly outweighing that of X chromosomes.

**Figure 6:**
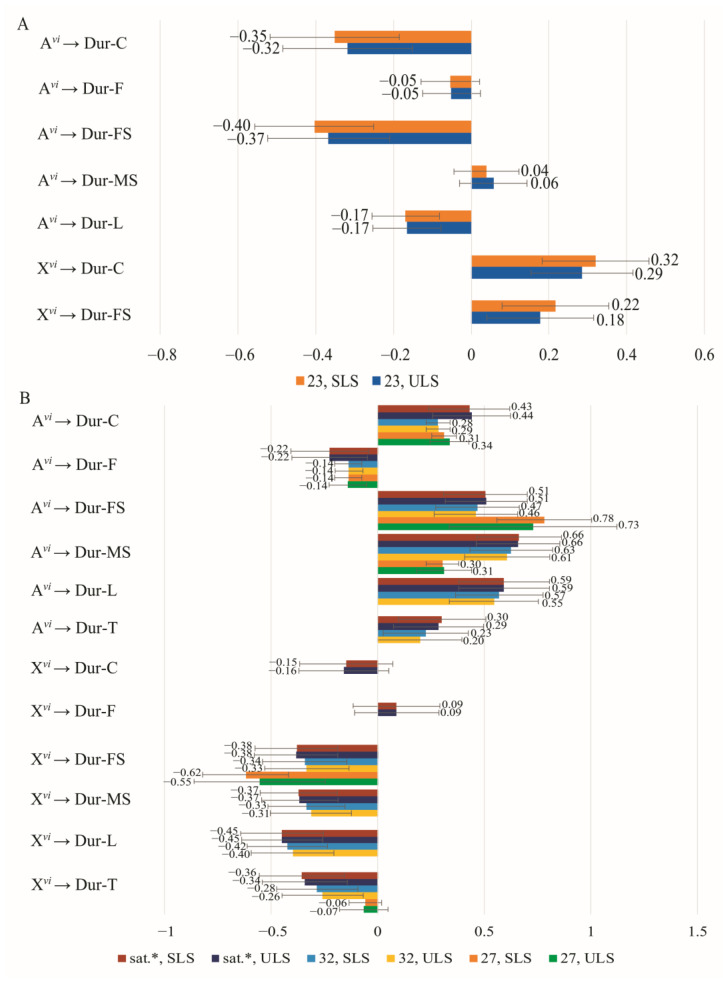
Effect of the proportion of *D. virilis* chromosomes in one courtship partner on the duration of courtship elements with the other partner having a constant *D. virilis* genotype: (**A**) Genotypes change in males while females have a *D. virilis* genotype (Group 1); (**B**) Genotypes change in females while males have a constant *D. virilis* genotype (Group 3).

As conspecificity increases in pairs with a constant *D. virilis* genotype of one partner, the standardized regression coefficients for the relationship between the proportion of *D. virilis* chromosomes and the duration of courtship elements are slightly lower in males compared to females (Figure 6A,B). The sets of courtship elements exhibit significant differences between males and females, particularly for elements influenced by X chromosomes. Additionally, the identical elements often differ in the sign of the regression coefficient.

As the heterospecificity of partners increases, the effect of male autosomes results in longer circling and female singing durations (Figure 6A). Conversely, in females under similar conditions, autosomes decrease the duration of these elements, as well as male singing and licking durations (Figure 6B). In males, conspecific X chromosomes extend the duration of circling and licking, whereas, in females, an increase in partner conspecificity leads to decreased durations of male and female singing, licking, and tapping influenced by X chromosomes.

Thus, the effects of *D. virilis* X chromosomes and autosomes on courtship elements exhibit notable differences between males and females. These differences manifest in specific latency and duration patterns, encompassing a repertoire of courtship elements that display a dependence on the species-specificity of X chromosomes and autosomes, with effects that are opposite in sign. However, some courtship elements demonstrate a similar impact of female and male genotypes on element latency. For instance, in pairs with a constant *D. americana* genotype (Groups 2 and 4), similar effects of autosomes on female singing latency and X chromosomes on male singing and licking latency were observed. Likewise, in pairs with a constant *D. virilis* genotype, similar influences of autosomes on circling and following latency were observed. Nonetheless, an important regularity should be highlighted: as the proportion of *D. virilis* chromosomes increases, along with an increase in heterospecificity (in pairs with a constant *D. americana* partner), the effect size of the male genotype becomes significantly larger than that of the female genotype. Conversely, when the proportion of *D. virilis* chromosomes increases, accompanied by an increase in conspecificity (in pairs with a constant *D. virilis* partner), the opposite effect is observed, with a more substantial contribution from the female genotype. These disparities in the effect size of the *D. virilis* genotype in males on courtship elements, observed in pairs with a *D. virilis* female (minimum) and in pairs with a *D. americana* female (maximum), may be attributed to a nonlinear increase in the genotype’s effect as pair heterospecificity rises.

### 3.5. Effect of Courtship Elements on Copulation Efficiency

The estimates of courtship-element latencies’ influence on copulation latency, obtained from the selected models using the ULS and SLS methods, consistently exhibit the same sign and are closely aligned in value, as shown in Table 5 and Table 6. Differences in significance can be attributed to the higher standard error estimated by the SLS method, resulting in the absence of a statistically significant difference in the regression coefficient from zero. It is important to note that all regression coefficients are presented in a standardized form, enabling meaningful comparisons among them.

In experimental Group 2, where females have a constant *D. americana* genotype and were tested with nonconstant genotype males, a significant negative correlation is observed between copulation latency and the latencies of tapping, following, and male singing (Table 5). Conversely, the latency of licking shows a positive correlation with copulation latency. This indicates that an earlier initiation of copulation is associated with an accelerated occurrence of licking and a delayed onset of tapping, following, and male singing. In Group 1, consisting of *D. virilis* females, copulation latency demonstrates a weaker negative relationship with the latency of following and a positive relationship with male singing latency. When experiments involving males with the *D. americana* genotype (Group 4) are combined into a single group, the models reveal a modest negative relationship between copulation latency and the latencies of circling and following, as well as a positive relationship between copulation latency and male singing latency. Experimental Group 3, which includes *D. virilis* males, exhibits a mild negative correlation between copulation latency and the latency of the following, a more pronounced negative correlation between copulation latency and licking latency, and a positive correlation between copulation latency and male singing latency.

The relationship between courtship-element duration and copulation duration is much more pronounced (Table 6). In a series of experiments with *D. americana* females (Group 2), it was found that only the duration of licking demonstrated a negative correlation with copulation duration, whereas circling, following, and male singing exhibited a positive correlation. In Group 1 experiments with *D. virilis* females, copulation duration is positively correlated with male singing duration and negatively correlated with licking duration. In the group of experiments involving males with the *D. americana* genotype (Group 4), all six available elements influence copulation duration (Model 29). The duration of tapping and male singing demonstrates a negative correlation with copulation duration, while the duration of licking, circling, following, and female singing displays a positive correlation with copulation duration. It should be noted that model 26 (Table 6) confirms the effect of tapping and licking while maintaining the regression’s sign, thereby indicating the highest reliability of these two elements and their relationship with copulation duration. Experimental Group 3, involving males with a constant *D. virilis* genotype, presents a different set of relationships between courtship-element duration and copulation duration across different models. Considering the elements presented in most models, which coincide with the regression’s sign, a negative relationship is observed between copulation duration and tapping duration, and a positive relationship is observed between copulation duration and licking duration, circling duration, and female singing duration.

The results obtained indicate significant differences in the effects of the genotype of males and females on courtship traits. The overall impact of genotype on the outcome of the courtship ritual differs between males and females. X chromosomes have a greater significance for males, while autosomes have a greater significance for females.

Minimal effects of the female genotype on the parameters of courtship elements were observed in tests with a male of constant *D. americana* genotype (Figure 3B and Figure 5B), while maximum effects of the female genotype were observed in tests with a *D. virilis* male (Figure 4B and Figure 6B).

These findings suggest that *D. americana* males exhibit lower sensitivity to the female genotype and display active behavior regardless of the female genotype. Consequently, they make a greater contribution to the courtship elements compared to females. Since most elements of the courtship ritual are demonstrated by the male, pairs with *D. americana* males showed the highest dependence of copulation success on the parameters of the courtship elements. On the other hand, *D. virilis* males exhibit a higher sensitivity to the female genotype, resulting in an increase in ritual violations with greater female heterospecificity and a decrease in the contribution of males to the parameters of the courtship ritual. As a result, the contribution of female partners increases, and the dependence of copulation effectiveness on the courtship elements decreases.

In tests with females of a constant genotype, the opposite pattern is observed. The effect of the male genotype in pairs with *D. americana* females (Figure 3A and Figure 5A) is higher than in pairs with *D. virilis* females (Figure 4A and Figure 6A), which can be attributed to the lower sensitivity of *D. americana* females to the male genotype. Consequently, in such pairs, there is a maximum dependence of copulation success on the parameters of the courtship elements.

In pairs with a *D. virilis* female, the standardized indicators of the male genotype’s influence reach minimal values, indicating a decrease in the male’s contribution to the parameters of courtship elements and a reduced effect on copulation efficiency.

The results presented in this section serve as quality control for the obtained estimates. Previously published experimental data on the impact of each Drosophila courtship element on copulation efficiency in conspecific and heterospecific tests, including estimates obtained for the species used in this study and related species, are available. In the Discussion section, we will compare our estimates obtained through structural equation modeling with the results of earlier behavioral experiments.

## 4. Discussion

Precopulatory isolation mechanisms encompass geographic, ecological, seasonal, mechanical, and ethological isolation. The first three factors involve the spatial, temporal, or ecological separation of closely related species, while mechanical isolation is associated with anatomical differences in the male and female reproductive organs that hinder successful copulation. Among these mechanisms, ethological isolation is the most effective in restricting interspecific mating. The final two isolation mechanisms, namely (1) divergence in mating organ morphology and selective mating and (2) the development of species-specific behavioral patterns, are driven by sexual selection. Intrasexual competition for optimal mating partners leads to the emergence of distinct markers that facilitate the assessment of genetic compatibility between individuals. These markers are examined during courtship and can manifest as visual, acoustic, and/or chemical stimuli that are genetically determined.

### 4.1. The Effect of Sex Chromosomes and Autosomes on the Efficiency of Copulation

Our investigation focused on assessing the influence of *D. virilis* X chromosomes and autosomes on copulation efficiency and the expression of courtship elements within the context of the *D. americana* genome. The *D. americana* genome includes the neo-X chromosome, which results from the fusion of the X and fourth chromosomes [32,33], and the neo-Y chromosome, which is represented by a fourth chromosome in addition to the normal Y chromosome in males [34,35,36]. Two aspects related to the neo-X and neo-Y chromosomes should be considered when evaluating the impact of sex chromosomes and autosomes on courtship behavior and copulation. The first concern relates to the potential bias in the weight coefficients of *D. virilis* X chromosomes and autosomes in males and females from the F_2_ and backcrosses due to chromosome segregation disruption in F_1_ individuals and selective elimination of certain genotypes in subsequent generations. In our study, as there were no significant differences in offspring survival between control crosses and F_1_ hybrids, we can disregard the issue of estimate distortion due to shifts in chromosome population composition. The second issue arises from the rearrangement of the fourth chromosome in *D. americana*, where it fuses with an X chromosome, resulting in a symmetrical distribution of the fourth chromosome of *D. virilis* with its X or Y chromosomes. Chromosome segregation distortion is commonly observed in the second generation and offspring from backcrosses, while the overall proportion of chromosomes from both species in the offspring remains unchanged. In our proposed models, we determine the regression coefficients based on the weight coefficients of both species’ chromosomes, corresponding to their proportion in the offspring. The influence of segregation distortion in this case causes a shift in the estimates of homozygotes and heterozygotes for the fourth chromosome, favoring homozygotes by approximately 25%. Consequently, this suggests a contribution of this autosome to the obtained estimates of the X chromosome’s impact.

All estimates of the effect of the proportion of *D. virilis* chromosomes are presented in a standardized form, allowing for comparisons both within each experimental group and between the groups, for both sex chromosomes and autosomes. Given that copulation success tends to decrease with increasing heterospecificity in pairs, we hypothesized that, overall, the proportion of *D. virilis* chromosomes would have a negative impact on copulation latency and a positive impact on copulation duration in pairs with a *D. virilis* partner (either male or female). As anticipated, we observed the opposite pattern for pairs with a *D. americana* partner: the overall effect of *D. virilis* chromosomes on copulation latency was positive, while it was negative for copulation duration. Furthermore, we found that as the genetic similarity between mating partners increased, the latency decreased, and the copulation duration increased, suggesting more thorough insemination of the female.

Estimates based on the absolute values of the male genotype’s effect on copulation success predominantly indicate the influence of sex chromosomes, while the effect of the female genotype is primarily associated with autosomes. These differences can be explained by models of sex chromosome and autosomal evolution rates [37,38], which consider constraints on the expression of sex-specific genes, the accumulation of substitutions in males and females, and the degree of allele dominance. It is important to note that the rate of chromosome evolution corresponds to the fixation rate of alleles linked to these chromosomes, thereby influencing the overall contribution of chromosomes to diverging traits. Studies on Drosophila have shown at least equal rates of variability accumulation in males and females or a higher rate in males [39,40]. According to the models [37,38], we can expect an underrepresentation of differentially expressed genes located on the X-chromosome in females, regardless of the degree of their dominance, and the same underrepresentation in males if sex-limited genes are predominantly dominant. In other words, in pairs where the male has a constant genotype, Aut*^D. virilis^* should consistently make a predominant contribution to copulation efficiency and the expression of courtship elements in females. Similarly, in pairs where the female has a constant genotype, X*^D. virilis^* should make a predominant contribution in males, assuming there is significant recessiveness of alleles of genes differentially expressed in males. The contributions of X chromosomes in males and autosomes in females, being the most significant, determine the overall effects of chromosomes on copulation efficiency and courtship elements.

The observed influence of female autosomes on copulation efficiency, which is mediated by rapidly evolving courtship elements, aligns well with the expected effects according to evolutionary rate models. However, the impact of the male X chromosome is less evident. In the genus Drosophila, there is a notable scarcity of genes with a male-biased expression on the X chromosome, likely due to a transfer of such genes from the X chromosome to autosomes [41]. The preferential dominance of alleles could explain the higher rate of mutation accumulation on autosomes in male-biased genes [38]. However, it does not account for the selective reduction of copies of such genes on the X chromosome.

As previously mentioned, recessive alleles of male-biased genes are rapidly fixed on the X chromosome. This suggests that there is an accumulation of adaptively significant variability in the models of sex chromosomes and autosomal evolution. Furthermore, the conclusions regarding the impact of allele dominance and the difference in mutation rates between males and females on the rate ratios are also applicable to the accumulation of mutation load. It is important to note that recessive mutations predominantly contribute to the genetic variation of traits that determine fitness. These mutations can be categorized into three types of alleles:

(i) Recently emerged deleterious alleles that have low frequencies and lead to reduced fitness;
(ii) Neutral and slightly deleterious alleles that are in equilibrium;
(iii) Alleles with intermediate frequencies that are maintained by opposing or frequency-dependent selection [42,43].

Estimates of the variability proportion for individual fitness components, conducted on populations of *D. melanogaster*, have revealed a substantial contribution, of at least 50%, from newly emerged alleles with deleterious effects [44,45,46]. Experiments on inbred populations of Drosophila and Mimulus [47,48,49] (method description: [50]) were carried out to assess the relative influence of the contribution of neutral and slightly deleterious mutations. These experiments provide compelling evidence for the significant effect of alleles that constitute a pool of constant genetic variability and occur at intermediate frequencies. Hence, the accelerated accumulation of recessive alleles on the X chromosome, linked to detrimental or slightly deleterious effects approaching neutrality in terms of viability, leads to the degradation of male-biased X-linked genes in the presence of paralogs or functional homologs on autosomes.

It can be assumed that the demasculinization of the X chromosome would result in a reduction of its contribution to traits selectively expressed in males. However, this conclusion is not absolute. In *D. melanogaster*, *D. simulans*, *D. ananassae*, and *D. mojavensis*, as well as on Muller A elements, there is a consistent underrepresentation of male-biased genes on the X chromosome, which has persisted and evolved for millions of years, comprising 30–43% [41]. The underrepresentation of these genes on the neo-X chromosome of *D. pseudoobscura*, formed through the fusion of Muller A and Muller D elements 8–12 million years ago, is 37%. If there were constant negative selection and erosion of male-biased genes on the X chromosome, their proportion on the “old” X chromosomes would be significantly lower. However, certain factors seem to restrict the erosion of some male-biased genes on the X chromosome, resulting in the demasculinization process reaching a plateau at approximately 70% of the expected level. These factors likely include rapidly evolving genes that play a role in prezygotic isolation barriers and are directly affected by sexual selection. Previous studies on mating behavior variability in *D. virilis* males with conspecific and heterospecific females (*D. americana texana* and *D. novamexicana*) demonstrated the prominent role of the X chromosome in male behavior during the tapping stage of courtship with heterospecific females [16]. Apart from courtship traits, these factors may also involve morphological and physiological characteristics associated with fertilization.

For instance, a study investigating the contribution of sex chromosomes and autosomes to the species-specific shape of the copulatory apparatus in *D. virilis* and *D. lummei* males revealed a disproportionately higher contribution of the X chromosome to the variability of shape traits (partial eta-squared) than expected [51].

### 4.2. Variability of the Courtship Structure

A comparative analysis of courtship structure variability in all tested variants with different degrees of conspecific and heterospecific pairs revealed a general similarity in structure. 

The longest courtship elements were tapping and licking, which aligns with previously reported findings in closely related species of the *virilis* group and other Drosophila species [2,27,28,52]. Typically, the courtship ritual is initiated with this pair of elements, followed by the addition of acoustic signals from both the male’s singing and the female’s response, usually coinciding with licking and tapping. The second pair of elements had a shorter duration compared to the first pair but exceeded the duration of the remaining elements [28,52,53]. Elements such as following, circling, and copulation attempts were characterized by shorter durations and occurred later in the courtship sequence if present. Previous studies have shown that circling behavior is more typical in *D. americana* compared to *D. virilis* [52]. Additionally, *D. americana* males display simultaneous tapping and licking, while *D. virilis* males initiate the second element somewhat later [52].

The analysis of courtship behavior in heterospecific pairs revealed differences in the courtship elements employed by *D. virilis* and *D. americana* males. Typically, *D. americana* males exhibited a complete courtship ritual, while *D. virilis* males ceased courting *D. americana* females at the tapping stage in one-third of the tests. This finding aligns with previous studies on heterospecific crosses involving sibling species of the *virilis* group [12,28,52]. Specifically, a significant proportion of *D. virilis* males refused to court females of *D. lummei*, *D. americana*, and *D. littoralis* after a brief tapping, whereas males of these species actively courted *D. virilis* females. This asymmetric change in the courtship structure during reciprocal heterospecific tests with specific combinations of sibling species was characterized by a considerable reduction in the licking stage, a substantial decrease in the duration of all courtship elements, and a significant decline in the number of copulations in one direction. Conversely, a significant increase in the duration of key courtship elements was observed in the other direction. For instance, this pattern was observed in the courtship behavior of *D. virilis* males towards females of *D. lummei*, *D. americana*, *D. novamexicana*, and *D. littoralis*, while the courtship ritual of *D. americana*, *D. novamexicana*, and *D. littoralis* males towards *D. virilis* females exemplified the second direction. These observations indicate the high significance of the chemical communication channel in species recognition during mate selection in *D. virilis*.

Many Drosophilidae species possess contact chemoreceptors on the tarsi of their front legs, maxillary palps, and proboscis [54]. During tapping and licking behaviors, males detect nonvolatile pheromones on the sternites’ surface of females through taste-receptor neurons [55]. Studies have demonstrated that *D. virilis* males can readily distinguish females of their own species from those of sibling species such as *D. americana americana*, *D. americana texana*, and *D. novamexicana*. Conversely, males of *D. americana americana*, *D. americana texana*, and *D. novamexicana* struggle to differentiate *D. virilis* females from females of their own species during the tapping stage [56]. Evidently, the chemical signal received by *D. virilis* males either ceases or strongly inhibits courtship, as they recognize heterospecific females as alien. This results in a complete cessation of courtship or a significant reduction in the ritual, leading to a very low copulation frequency. On the other hand, *D. americana* males do not perceive *D. virilis* females as strangers; however, there is a breakdown in the signal exchange between partners, leading to a substantial increase in the duration of the tapping and licking stage. It is possible that in these instances, *D. virilis* females do not recognize heterospecific males (“hesitation”), prompting the males to exert additional effort to ensure courtship culminates in copulation. Clearly, these differences in courtship behavior between *D. virilis* and *D. americana* are genetically determined. The presence or absence of species-specific alleles will result in corresponding phenotypic manifestations that influence the courtship-ritual structure, either aligning it with the conspecific variant (*D. virilis*) or deviating from it.

In addition to the existing differences and disruptions in the courtship-ritual program between *D. virilis* and *D. americana*, there were also changes in the proportion of successfully copulating pairs. Among all experiments, the lowest percentage of copulating pairs was observed in heterospecific tests involving *D. virilis* + *D. americana* in both testing directions. It is noteworthy that during the designated observation period, the smallest number of copulations (only one copulation per 30 tested pairs) was consistently observed in the ♀ *D. americana* + ♂ *D. virilis* direction, resulting in all hybrids produced in this direction. On the other hand, in the reciprocal direction ♀ *D. virilis* + ♂ *D. americana*, approximately one-third of the pairs (11 pairs out of 30) successfully copulated. However, it is important to note that, despite this relatively higher copulation rate compared to the other reciprocal variant, the females did not produce viable offspring. This can be explained by the presence of postcopulatory isolation, as previously documented by various authors [21,22,57].

It is important to note that the increased duration of nearly all courtship elements is attributed to disruptions in signal exchange through the acoustic communication channel [29]. The acoustic duet between the female and male is known to play a crucial role in the successful progression of the courtship ritual in *D. virilis* [7] and any disturbance to this duet leads to changes in the courtship structure. For instance, *D. virilis* males courting *D. americana* females exhibited a reduction in the duration of mating songs, indicating decreased courtship intensity due to early recognition of the heterospecific female during the tapping stage. Conversely, courtship from a *D. americana* male increased the duration of female singing in *D. virilis*. These findings align with previous studies demonstrating that females exhibit active singing not only in conspecific tests but also in heterospecific tests where males engage in prolonged licking and singing [7,28]. Female singing is likely a stimulus that encourages males to persist in their courtship but does not directly contribute to the successful completion of the ritual. Based on the observation that *D. americana* males attempted copulation after an extended courtship period in heterospecific tests, it can be concluded that the low percentage of successful copulations was determined by the final choice of the female during the last stage of courtship.

## 5. Conclusions

We have demonstrated that autosomes predominantly contribute to courtship elements implemented by females while X chromosomes play a significant role in elements implemented by males. *D. americana* males and females exhibit lower sensitivity to mate stimuli compared to *D. virilis*. The impact of the *D. virilis* genotype in females paired with *D. americana* males significantly surpasses its effect in males paired with *D. americana* females. However, these differences are notably reduced in pairs with the reference *D. virilis* genotype and there is little disparity in the duration of courtship elements. This leads to an asymmetric pattern in the influence of the *D. virilis* genotype on courtship behavior between males and females in reciprocal tests. The effect primarily manifests in changes in the duration and latency of elements such as male singing, tapping, and licking, which are associated with acoustic and chemical communication channels. This suggests a nonlinear increase in the impact of the *D. virilis* genome on deviations from genetically determined behavioral programs as its proportion increases in males relative to females. It is possible that sexual selection plays a role in maintaining sex-specific male genes responsible for courtship partner recognition and stimulation on the X chromosome.

## Figures and Tables

**Table 1:** The variants of the no-choice behavioral tests were categorized based on the consistent parental genotype of one partner and the escalation in heterospecificity of the other partner.

Female	Male	Chromosome Composition *	X^Vi^/A^Vi^ (%)	Sum Cop.	Fisher’s Exact Test, p
**Group 1 (♀ *D. virilis*–♂ *D. virilis* → *D. americana*)**					
♀ *D. virilis*	♂ *D. virilis*	Y^Vi^/X^Vi^; A^Vi^/A^Vi^	100/100	30	
♀ *D. virilis*	♂ F_B_ (♀♀ *D. virilis* × ♂♂ F_1_)	Y^Vi^/X^Vi^; A^(Am,Vi)^/A^Vi^	100/75	29	0.5000
♀ *D. virilis*	♂ F_B_ (♀♀ F_1_ × ♂♂ *D. virilis*)	Y^Vi^/X^(Am,Vi)^; A^(Am,Vi)^/A^Vi^	50/75	28	0.2458
♀ *D. virilis*	♂ F_2_	Y^Vi^/X^(Am,Vi)^; A^Am^/A^Vi^	50/50	***25***	***0.0261***
♀ *D. virilis*	♂ F_1_	Y^Vi^/X^Am^; A^Am^/A^Vi^	0/50	***23***	***0.0053***
♀ *D. virilis*	♂ *D. americana*	Y^Am^/X^Am^; A^Am^/A^Am^	0/0	***11***	***0.0000***
**Group 2 (♀ *D. americana*–♂ *D. americana* → *D. virilis*)**					
♀ *D. americana*	♂ *D. americana*	Y^Am^/X^Am^; A^Am^/A^Am^	0/0	29	
♀ *D. americana*	♂ F_B_ (**♀♀** *D. americana* × ♂♂ F_1_)	Y^Vi^/X^Am^; A^(Am,Vi)^/A^Am^	0/25	28	0.5000
♀ *D. americana*	♂ F_1_	Y^Vi^/X^Am^; A^Am^/A^Vi^	0/50	27	0.3060
♀ *D. americana*	♂ F_B_ (**♀♀** F_1_ × ♂♂ *D. americana*)	Y^Am^/X^(Am,Vi)^; A^(Am,Vi)^/A^Am^	50/25	24	0.0514
♀ *D. americana*	♂ F_2_	Y^Vi^/X^(Am,Vi)^; A^Am^/A^Vi^	50/50	27	0.3060
♀ *D. americana*	♂ *D. virilis*	Y^Vi^/X^Vi^; A^Vi^/A^Vi^	100/100	***1***	***0.0000***
**Group 3 (♀ *D. virilis* → *D. americana*–♂ *D. virilis*)**					
♀ *D. virilis*	♂ *D. virilis*	X^Vi^/X^Vi^; A^Vi^/A^Vi^	100/100	30	
♀ F_B_ (♀♀ F_1_ × ♂♂ *D. virilis*)	♂ *D. virilis*	X^Am^/X^3Vi^; A^(Am,Vi)^/A^Vi^	75/75	29	0.5000
♀ F_B_ (♀♀ *D. virilis* × ♂♂ F_1_ )	♂ *D. virilis*	X^Am^/X^Vi^; A^(Am,Vi^/A^Vi^	50/75	28	0.2458
♀ F_2_	♂ *D. virilis*	X^Am^/X^3Vi^; A^Am^A^Vi^	75/50	28	0.2458
♀ F_1_	♂ *D. virilis*	X^Am^/X^Vi^; A^Am^A^Vi^	50/50	29	0.5000
♀ *D. americana*	♂ *D. virilis*	X^Am^/X^Am^; A^Am^/A^Am^	0/0	***1***	***0.0000***
**Group 4 (♀ *D. americana* → *D. virilis*–♂ *D. americana*)**					
♀ *D. americana*	♂ *D. americana*	X^Am^/X^Am^; A^Am^/A^Am^	0/0	29	
♀ F_B_ (♀♀ *D. americana* × ♂♂ F_1_ )	♂ *D. americana*	X^Am^/X^Am^; A^(Am,Vi)^/A^Am^	0/25	24	0.0514
♀ F_B_ (♀♀ F_1_ × ♂♂ *D. americana*)	♂ *D. americana*	X^3Am^/X^Vi^; A^(Am,Vi)^/A^Am^	25/25	***23***	***0.0262***
♀ F_1_	♂ *D. americana*	X^Am^/X^Vi^; A^Am^/A^Vi^	50/50	***17***	***0.0002***
♀ F_2_	♂ *D. americana*	X^Am^/X^3Vi^; A^Am^/A^Vi^	75/50	25	0.0973
♀ *D. virilis*	♂ *D. americana*	X^Vi^/X^Vi^; A^Vi^/A^Vi^	100/100	***11***	***0.0000***

*D. virilis* and *D. americana* were the parental species, with F_1_ as the first generation of interspecific hybrids, F_2_ as the second generation of interspecific hybrids, and F_B_ as the offspring from backcrossing the first-generation hybrids with one of their parents. We conducted four groups of behavioral experiments, where one of the partners gradually progressed from a conspecific to a completely heterospecific genotype while the other partner’s genotype remained constant (1—**♀** *D. virilis*, 2—**♀** *D. americana*, 3—♂ *D. virilis*, 4—♂ *D. americana*). Within each group, the pairs were sorted in order of the increasing heterospecificity of the partner with a nonconstant genotype. Conspecific and completely heterospecific tests were repeated in two groups. “Sum cop.” represents the number of successful copulations among the 30 tested pairs. “Chromosome composition *” and “X^Vi^/A^Vi^ (%)” indicate the composition and percentage of *D. virilis* X chromosomes and autosomes in a partner with a changing chromosome composition (genotypes) within the group of experiments. Fisher’s exact test was used to compare the proportion of copulating pairs in a given experiment variant relative to the conspecific one.

**Table 2:** General variability of courtship traits in all experimental groups.

Traits	N	Mean	Median	Min.	Max.	Lower Quartile	Upper Quartile
Latency							
Following	600	1175.5	1801.0	0	1801	15.5	1801.0
Tapping	600	5.0	0.0	0	1801	0.0	0.0
Licking	600	77.0	2.0	0	1801	1.0	6.0
Male singing	600	145.5	4.5	0	1801	2.0	18.0
Circling	600	1119.1	1801.0	0	1801	33.0	1801.0
Copulation attempt	600	1375.6	1801.0	2	1801	1722.0	1801.0
Copulation	600	439.8	44.0	2	1801	9.0	477.0
Female singing	600	42.3	2.0	0	1801	0.0	7.0
Duration							
Following	600	4.4	0.0	0	107	0.0	3.0
Tapping	600	45.4	16.0	0	896	7.0	42.0
Licking	600	33.6	10.0	0	665	4.0	27.0
Male singing	600	11.9	5.0	0	210	3.0	13.0
Circling	600	2.9	0.0	0	108	0.0	3.0
Copulation attempt	600	4.4	0.0	0	204	0.0	0.5
Copulation	600	125.2	142.0	0	380	97.0	170.0
Female singing	600	11.7	4.0	0	435	2.0	12.0

N—the total number of the tested pairs; latency and duration are given in seconds.

**Table 3:** Selection of optimal models for the analysis of the influence of sex chromosomes and autosomes on courtship traits in experimental groups.

Constant Mate Genotype	Copulation Parameters	Model	Parameters	df	C	C − df	AIC	BCC	BIC	C/df
**♀ *D. virilis* (Group 1)**	latency	**14**	23	32	11.72	−20.28	**57.72**	**60.74**	**131.16**	0.37
		**16**	25	30	9.12	**−20.88**	59.12	62.39	138.94	**0.3**
**♀ *D. americana* (Group 2)**		**20**	23	22	4.54	−17.46	**50.54**	**53.26**	**123.97**	**0.21**
**♀ *D. virilis* (Group 1)**	duration	**23**	31	14	5.51	**−8.49**	**67.51**	**71.18**	**166.49**	0.39
**♀ *D. americana* (Group 2)**		**7**	28	17	61	44	117	120	**206**	**4**
		**23**	31	14	17	3	**79**	**83**	**178**	**1**
**♂ *D. virilis* (Group 3)**	latency	**23**	31	14	20.21	6.21	**82.21**	**85.87**	181.19	1.44
		**25**	33	12	16.81	4.81	82.81	86.71	188.18	**1.4**
**♂ *D. americana* (Group 4)**		**19**	27	18	4.99	**−13.01**	58.99	62.19	145.2	0.28
		**26**	34	11	2.54	−8.46	70.54	74.56	179.1	**0.23**
**♂ *D. virilis* (Group 3)**	duration	**27**	35	10	41.02	31.02	111.02	115.16	**222.77**	**4.1**
		**32**	40	5	24.9	19.9	104.9	109.63	232.61	4.98
		**Sat**	45	0	0	0	**90**	**95.33**	233.68	
**♂ *D. americana* (Group 4)**		**26**	35	10	2.55	**−7.45**	**72.55**	**76.69**	**184.3**	0.25

The minimum values of the information criteria are in bold with underlining. **Model**: the formal number of the model in the list of the evaluated models. **Parameters**: the number of parameters to be evaluated in the model. **df:** degrees of freedom. **C:** minimum value of the residual function. **C—df, AIC—Akaike information criterion, BCC—the Browne–Cudeck criterion, BIC—Bayesian information criterion, C/df**: information criteria. **p**: probability of a poor fit between the correct and chosen models. **Sat** (saturated): base model.

**Table 4:** Indirect influence of the X chromosome and autosomes on copulation latency and duration in the experimental groups.

Copulation Parameters	Constant Partner Genotype	Changing Partner Genotype	Effect *				Method	Model
			X^Vi^	X^Vi^ s.e.	Au^Vi^	Au^Vi^ s.e.		
Latency	♀ *D. virilis* (Group 1)	♂♂ vi → am	**−0.427**	**0.07**	−0.06	**0.03**	ULS	16
			**−0.433**	**0.071**	−0.06	**0.03**	ULS	14
	♀ *D. americana* (Group 2)	♂♂ am → vi	**1.775**	**0.627**	**−1.233**	**0.759**	ULS	20
Duration	♀ *D. virilis* (Group 1)	♂♂ vi → am	0	-	**0.531**	**0.068**	ULS	23
	♀ *D. americana* (Group 2)	♂♂ am → vi	**−0.725**	-	**0.022**	-	ULS	23
Latency	♂ *D. virilis* (Group 3)	**♀♀** vi → am	−0.03	0.259	**−0.65**	0.230	ULS	23
			**0.54**	0.590	**−1.12**	0.578	SLS	23
			**2.16**	0.93	**−2.69**	0.93	ULS	25
			**1.55**	1.518	**−2.12**	1.513	SLS	25
	♂ *D. americana* (Group 4)	**♀♀** am → vi	0.049	0.206	**0.302**	0.23	ULS	26
Duration	♂ *D. virilis* (Group 3)	**♀♀** vi → am	**−0.455**	0.144	**1.183**	0.158	ULS	Sat*
	♂ *D. americana* (Group 4)	**♀♀** am → vi	**−0.352**	-	−0.061	-	ULS	26
			**−0.369**	0.098	−0.043	0.126	SLS	26

vi: *D. virilis*, am: *D. americana*. Effect *—s.e.: standard error estimated by bootstrap. The values are given in a standardized form.

**Table 5:** Relationship between courtship-element latencies and copulation latency.

Fixed Mate Genotype; Model; Method	Latency					
	Tapping	Licking	Male Singing	Circling	Following	Female Singing
♀ *D. virilis* (Group 1); 16; ULS			**0.56**		**−0.248**	
s.e.			0.057		0.061	
♀ *D. virilis* (Group 1); 16; SLS			**0.685**		**−0.288**	
s.e.			0.059		0.061	
♀ *D. americana* (Group 2); 20; ULS	**−2.306**	**2.935**	**−2.222**		**−1.738**	
s.e.	1.409	1.872	1.79		1.07	
♀ *D. americana* (Group 2); 20; SLS	**−1.393**	**2.739**	−2.228		−1.02	
s.e.	1.28	2.289	2.387		1.115	
♂ *D. virilis* (Group 3); 25; ULS		−0.618	**1.08**		**−0.246**	0.041
s.e.		0.784	0.793		0.109	0.217
♂ *D. virilis* (Group 3); 25; SLS		**−1.187**	**1.691**		**−0.407**	−0.141
s.e.		1.166	1.351		0.296	0.592
♂ *D. virilis* (Group 3); 23; ULS		−0.522	**0.957**		**−0.234**	0.111
s.e.		0.673	0.689		0.091	0.172
♂ *D. virilis* (Group 3); 23; SLS		−0.949	**1.375**		**−0.357**	0.066
s.e.		1.114	1.213		0.146	0.3
♂ *D. virilis* (Group 3); 15; ULS			**0.432**		**−0.233**	0.18
s.e.			0.069		0.061	0.069
♂ *D. virilis* (Group 3); 15; SLS			**0.477**		**−0.272**	0.264
s.e.			0.067		0.06	0.058
♂ *D. americana* (Group 4); 26; ULS	−1.344	1.544	−0.448	**−0.181**	**−0.751**	
s.e.	1.376	1.993	1.484	0.119	0.574	
♂ *D. americana* (Group 4); 19; ULS	−0.055		**0.461**	**−0.141**	−0.194	
s.e.	0.37		0.238	0.076	0.229	
♂ *D. americana* (Group 4); 19; SLS	−0.127		0.591	−0.131	−0.132	
s.e.	1.182		0.733	0.343	1.139	

Bold type indicates standardized regression coefficients that are significantly different from zero.

**Table 6:** Relationship between courtship-element duration and copulation duration.

Fixed Mate Genotype; Model; Method	Duration					
	Tapping	Licking	Male Singing	Circling	Following	Female Singing
♀ *D. virilis* (Group 1); 23; ULS		**−2.43**	**2.248**			
s.e.		0.744	0.748			
♀ *D. virilis* (Group 1); 23; SLS		**−2.588**	**2.404**			
s.e.		0.833	0.837			
♀ *D. americana* (Group 2); 23; SLS		**−8.402**	**8.591**			
s.e.		7.863	7.888			
♀ *D. americana* (Group 2); 7; ULS		**−6.754**	**5.688**	**2.921**	**0.811**	
s.e.		3.643	3.079	1.568	0.576	
♀ *D. americana* (Group 2); 7; SLS		**−11.405**	**9.432**	**4.975**	**1.674**	
s.e.		7.534	6.343	3.143	1.243	
♂ *D. virilis* (Group 3); base; ULS	**−4.124**	**4.79**	**−1.471**	**0.395**	**0.626**	−0.026
s.e.	1.07	1.954	1.235	0.247	0.201	0.137
♂ *D. virilis* (Group 3); base; SLS	**−4.739**	**5.992**	−2.118	0.366	**0.655**	0.001
s.e.	2.255	4.434	2.848	0.432	0.351	0.256
♂ *D. virilis* (Group 3); 32; ULS	**−3.019**	**2.544**		**0.416**	−0.081	**0.293**
s.e.	0.537	0.522		0.117	0.099	0.186
♂ *D. virilis* (Group 3); 32; SLS	**−3.164**	**2.718**		**0.386**	−0.074	**0.27**
s.e.	0.595	0.576		0.115	0.099	0.187
♂ *D. virilis* (Group 3); 27; ULS	**−1.786**	**1.37**		**0.325**	**−0.154**	**0.388**
s.e.	0.195	0.245		0.127	0.099	0.163
♂ *D. virilis* (Group 3); 27; SLS	**−1.876**	**1.425**		**0.325**	**−0.147**	**0.429**
s.e.	0.22	0.27		0.14	0.099	0.173
♂ *D. americana* (Group 4); 29; SLS	**−10.179**	**10.194**	**−2.691**	**0.992**	**0.667**	**1.498**
s.e.	4.635	4.887	1.849	0.62	0.545	0.999
♂ *D. americana* (Group 4); 26; SLS	**−6.982**	**6.759**	−1.616	0.623	0.423	1.101
s.e.	5.752	5.541	1.757	0.845	0.616	1.191

Bold type indicates standardized regression coefficients that are significantly different from zero.

## Data Availability

All data generated or analyzed during this study are included in the manuscript.

## Supplementary Materials

The following supporting information can be downloaded at: https://www.mdpi.com/article/10.3390/insects14070609/s1, Figure S1: The scheme of hybridization and behavioral tests. Figure S2: Variability of courtship elements in tests with increasing levels of courtship mate heterospecificity. Text S1. Variability of Courtship Elements in Courtship-Partner Pairs with Various Genotype Combinations. Table S1: Variability of the courtship elements in pairs ♀ *D. virilis* + ♂ *D. virilis*; Table S2: Variability of the courtship elements in pairs ♀ *D. virilis* + ♂ *D. americana*; Table S3: Variability of the courtship elements in pairs ♀ *D. virilis* + ♂ F1; Table S4: Variability of the courtship elements in pairs ♀ *D. virilis* + ♂ F_2_; Table S5: Variability of the courtship elements in pairs ♀ *D. virilis* + ♂ F_B_ (♀♀ *D. virilis* × ♂♂ F_1_); Table S6: Variability of the courtship elements in pairs ♀ *D. virilis* + ♂ F_B_ (♀♀ F_1_ × ♂♂ *D. virilis*); Table S7: Variability of the courtship elements in pairs ♀ *D. americana* + ♂ *D. virilis*; Table S8: Variability of the courtship elements in pairs ♀ *D. americana* + ♂ *D. americana*; Table S9: Variability of the courtship elements in pairs ♀ *D. americana* + ♂ F_1_; Table S10: Variability of the courtship elements in pairs ♀ *D. americana* + ♂ F_2_; Table S11: Variability of the courtship elements in pairs ♀ *D. americana* + ♂ F_B_ (♀♀ *D. americana* x ♂♂ F_1_); Table S12: Variability of the courtship elements in pairs ♀ *D. americana* + ♂ F_B_ (♀♀ F_1_ × ♂♂ *D. americana*); Table S13: Variability of the courtship elements in pairs ♀ F_1_ + ♂ *D. americana*; Table S14: Variability of the courtship elements in pairs ♀ F_1_ + ♂ *D. virilis*; Table S15: Variability of the courtship elements in pairs ♀ F_2_ + ♂ *D. virilis*; Table S16: Variability of the courtship elements in pairs ♀ F_2_ + ♂ *D. americana*; Table S17: Variability of the courtship elements in pairs ♀ F_B_ (♀♀ *D. virilis* × ♂♂ F_1_) + ♂ *D. virilis*; Table S18: Variability of the courtship elements in pairs ♀ F_B_ (♀♀ *D. americana* × ♂♂ F_1_) + ♂ *D. americana*; Table S19: Variability of the courtship elements in pairs ♀ F_B_ (♀♀ F_1_ × ♂♂ *D. virilis*) + ♂ *D. virilis*; Table S20: Variability of the courtship elements in pairs ♀ F_B_ (♀♀ F_1_ × ♂♂ *D. americana*) + ♂ *D. americana*; Table S21: The effect of the male genotype on variability of the courtship elements in pairs with *D. virilis* female; Table S22: The effect of the male genotype on variability of the courtship elements in pairs with *D. americana* female; Table S23: The effect of the female genotype on variability of the courtship elements in pairs with *D. virilis* male. Table S24: The effect of the female genotype on the variability of the courtship elements in pairs with *D. americana* male. Table S25: Results of the Mann-Whitney U Test. Table S26: Results of the Wald-Wolfowitz Runs Test. Table S27: Evaluation of model fitting for data sets pooled in accordance with male or female homology (Groups 1–4).
