# Supplementary material for: The Effect of Chromosomes on Courtship Behavior in Sibling Species of the Drosophila virilis Group
Source: Insects. 2023 Jul 5;14(7):609. doi: 10.3390/insects14070609 (PMC10380318; doi:10.3390/insects14070609)
Supplement: Supplementary file 1 [file insects-14-00609-s001.zip › insects-2445184-supplementary.pdf]

Figure S1. The scheme of crosses and courtship tests. P – parental species (D. americana (Am) and D. virilis (Vi)); F1 – the first generation of interspecies hybrids; F2 – the second generation of interspecies hybrids; Fb1, Fb3 – backcrosses F1 × D. virilis; Fb2, Fb4 – backcrosses F1 × D. americana. Colored frames designate four experimental groups: green – a female with constant genotype D. virilis (group 1); blue – a female with constant genotype D. americana (group 2); orange – a male with constant genotype D. virilis (group 3); violet – a male with constant genotype D. americana (group 4). Crosses are designated with "x", courtship interactions are designated with "+".

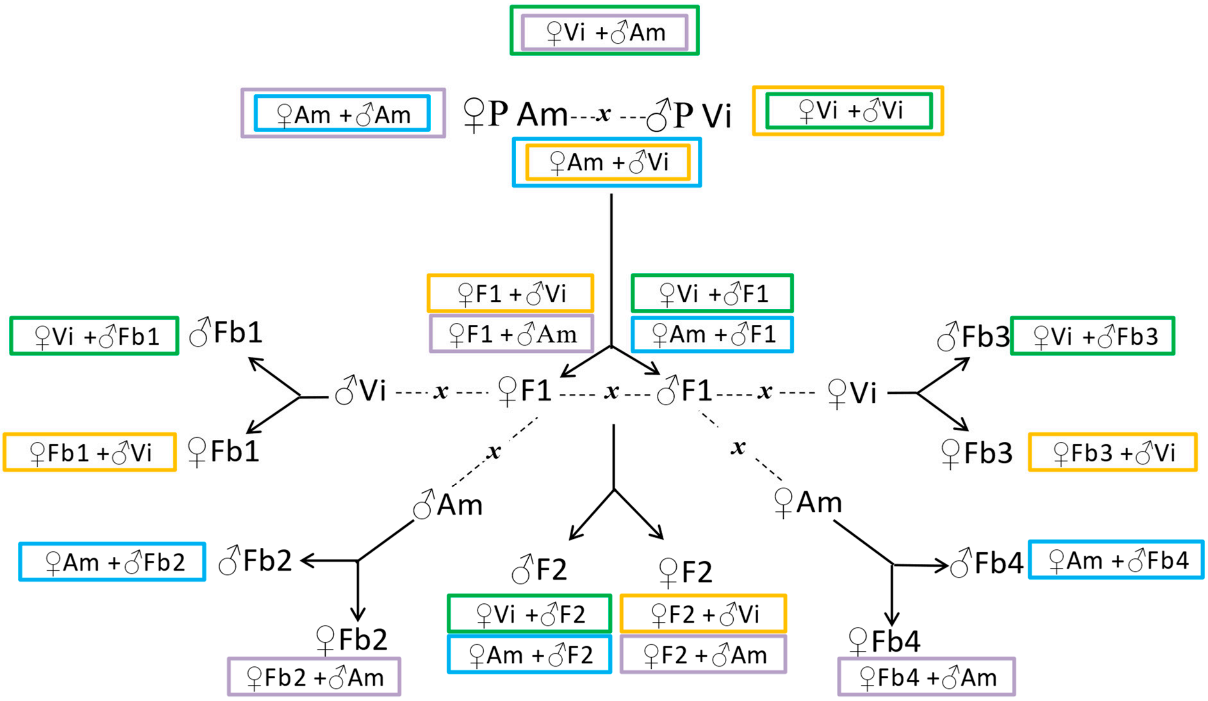

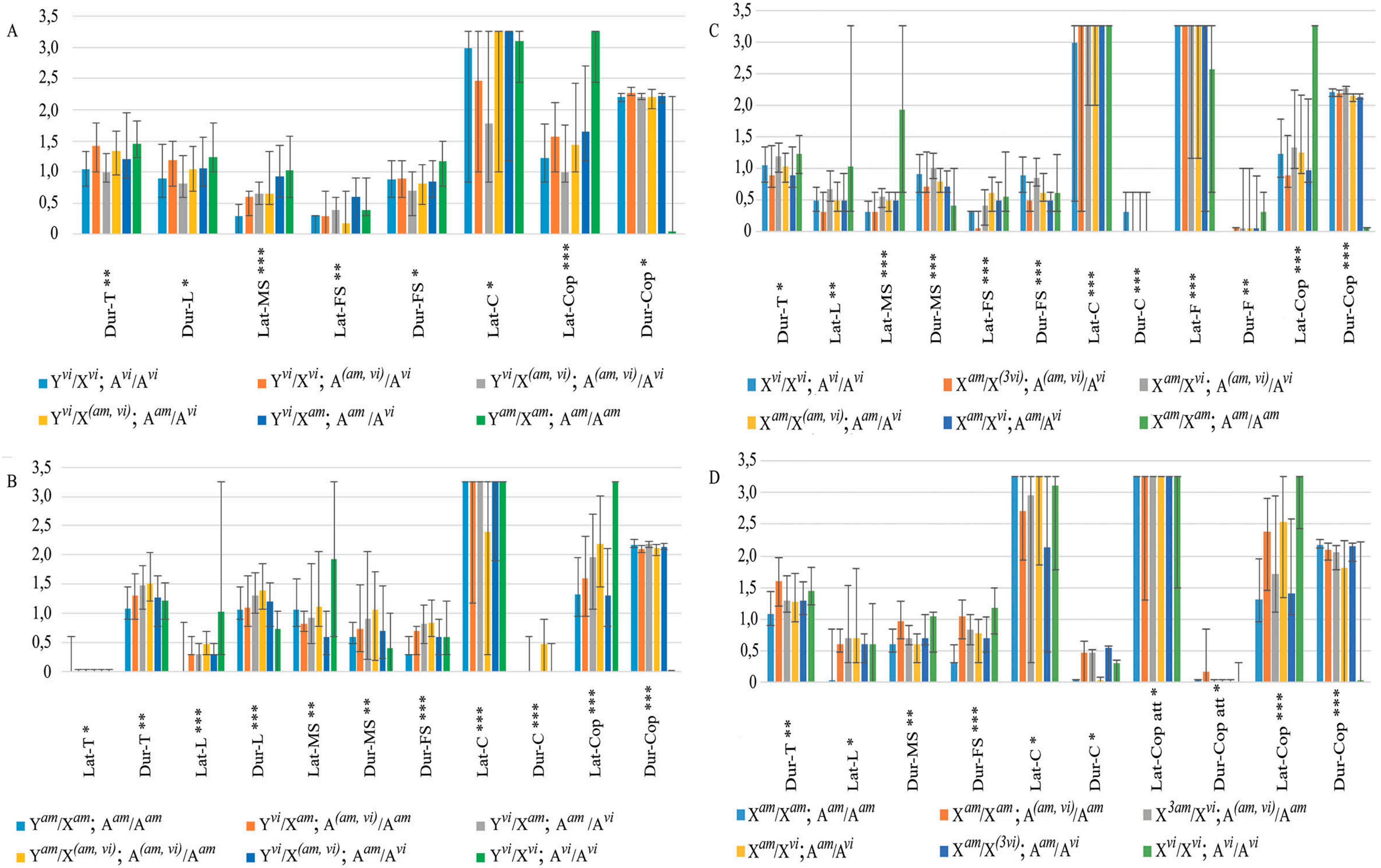
A
Dur-T **
Dur-L *
Lat-MS ***
Lat-FS **
Dur-FS *
Lat-C *
Lat-Cop ***
Dur-Cop *
Y^{vi}/X^{vi}; A^{vi}/A^{vi}
Y^{vi}/X^{vi}; $A^{(am,\ vi)}/A^{vi}$
$Y^{vi}/X^{(am,\ vi)}$; $A^{(am,\ vi)}/A^{vi}$
$Y^{vi}/X^{(am,\ vi)}$; A^{am}/A^{vi}
Y^{vi}/X^{am}; A^{am}/A^{vi}
Y^{am}/X^{am}; A^{am}/A^{am}
B
Lat-T *
Dur-T **
Lat-L ***
Dur-L ***
Lat-MS **
Dur-MS **
Dur-FS ***
Lat-C ***
Dur-C ***
Lat-Cop ***
Dur-Cop ***
Y^{am}/X^{am}; A^{am}/A^{am}
Y^{vi}/X^{am}; $A^{(am,\ vi)}/A^{am}$
Y^{vi}/X^{am}; A^{am}/A^{vi}
$Y^{am}/X^{(am,\ vi)}$; $A^{(am,\ vi)}/A^{am}$
$Y^{vi}/X^{(am,\ vi)}$; A^{am}/A^{vi}
Y^{vi}/X^{vi}; A^{vi}/A^{vi}
C
Dur-T *
Lat-L **
Lat-MS ***
Dur-MS ***
Lat-FS ***
Dur-FS ***
Lat-C ***
Dur-C ***
Lat-F ***
Dur-F **
Lat-Cop ***
Dur-Cop ***
X^{vi}/X^{vi}; A^{vi}/A^{vi}
$X^{am}/X^{(3vi)}$; $A^{(am,\ vi)}/A^{vi}$
X^{am}/X^{vi}; $A^{(am,\ vi)}/A^{vi}$
$X^{am}/X^{(am,\ vi)}$; A^{am}/A^{vi}
X^{am}/X^{vi}; A^{am}/A^{vi}
X^{am}/X^{am}; A^{am}/A^{am}
D
Dur-T **
Lat-L *
Dur-MS **
Dur-FS ***
Lat-C *
Dur-C *
Lat-Cop att *
Dur-Cop att *
Lat-Cop ***
Dur-Cop ***
X^{am}/X^{am}; A^{am}/A^{am}
X^{am}/X^{am}; $A^{(am,\ vi)}/A^{am}$
X^{3am}/X^{vi}; $A^{(am,\ vi)}/A^{am}$
X^{am}/X^{vi}; A^{am}/A^{vi}
$X^{am}/X^{(3vi)}$; A^{am}/A^{vi}
X^{vi}/X^{vi}; A^{vi}/A^{vi}

Figure S2. Variability of courtship elements in tests with increasing levels of courtship mate heterospecificity: (a) ♀ D. virilis + ♂ D. virilis → D. americana (Group 1); (b) ♀ D. americana + ♂ D. americana → D. virilis (Group 2); (c) ♂ D. virilis + ♀ D. virilis → D. americana (Group 3); (d) ♂ D. americana + ♀ D. americana → D. virilis (Group 4). X-axis: parameters of the courtship elements; Y-axis: log(10)-transformed estimates of latency and duration in seconds; whiskers designate upper and lower quartiles; * - $0.01 < p < 0.05$, ** - $0.001 < p < 0.01$, $p < 0.001$. Results of the Kruskal-Wallis test are presented in Table S21-S24.

Courtship elements that do not show significant differences within each group are not presented. Abbreviations: Latency – Lat; Duration – Dur; Tapping – T; Licking – L; Circling – C; Male song – MS; Female song – FS; Following – F; Copulation attempt - Cop att; Copulation – Cop.

Text S1. Variability of Courtship Elements in Courtship-Partner Pairs with Various Genotype Combinations

The position of partner genotypes in the graphs from left to right corresponds to an increase in the proportion of heterospecific chromosomes in the partner with a non-constant genotype. Variability values are only presented in the graphs for traits that exhibit marked differences in the analyzed groups. The results of comparing all traits across the four groups are presented in Tables S23-S26 in the supplementary materials.

Tapping (T - Tapping) is one of the key courtship elements in the *Drosophila virilis* species group. This element is usually the first to manifest itself during the courtship ritual in Drosophila of the *virilis* group and is often performed simultaneously with other courtship elements, such as licking. The duration of tapping is nearly uniform for both *D. virilis* and *D. americana* and averages 26 seconds. An increase in the proportion of the genome of another species leads to an increase in tapping duration. This tendency is strongly observed in the presence of a female *D. virilis* genotype (Group 1) and is contingent on the composition of the male chromosomes. A significant increase in tapping duration is observed both among males from backcrossing ♀ F_1 × ♂ *D. virilis* and *D. americana* males. Although there is a rise in the median value of tapping duration in pairs with F_2 and F_1 males, the increase's significance is not confirmed.

The trend is less pronounced with constant female and male *D. americana* genotypes in Groups 2 and 4, correspondingly. As the heterospecificity in pairs increases, a significant increase in the tapping duration is only discerned among partners from the backcrossing of ♂ *D. americana* × ♀ F_1. In Group 3, a transformation of the female genotype does not result in a notable rise in values against the constant male *D. virilis* genotype background. Furthermore, in one of the experiments, tapping duration decreases, specifically in the case of F_1 females.

The licking duration in *D. americana* is marginally higher than that in *D. virilis*. This may be one of the reasons for an increased licking duration with an increased proportion of the *D. americana* genotype in the male in pairs with *D. virilis* females with a constant genotype (Group 1) and, conversely, for a decrease in duration with an increased proportion of the *D. virilis* genotype in the male in pairs with *D. americana* females with a constant genotype (Group 2).

Noteworthy, there is a significant difference between pairs featuring *D. virilis* females with males from a ♀ *D. virilis* × ♂ F_1 backcross with minimal licking duration and pairs with *D. virilis* females and *D. americana* males (a totally heterospecific variant) with maximal licking duration. Furthermore, the minimum value of the licking duration in pairs with *D. americana* females (Group 2) is either absent or very minimal in entirely heterospecific pairs. Finally, the licking duration remains uninfluenced by the chromosome composition of females (Groups 3 and 4).

Significant variability concerns not only the licking duration but also the licking latency. Conspecific *D. americana* pairs display median latency values of zero. This suggests that in most pairs, licking is initiated from the beginning of the courtship, concurrently with tapping. Conversely, in conspecific *D. virilis* pairs, the licking latency is quite pronounced; licking is usually executed after the start of tapping, not concurrently.

In experiments involving both female and male *D. americana* with non-constant genotype partners (Groups 2 and 4), including those with the lowest proportion of the *D. virilis* genome, licking did not occur instantaneously with tapping but was rather delayed. Specifically, hybrids exhibit a courtship pattern that is characteristic of *D. virilis*. However, there are differences in the effect of a changing chromosome composition in males compared to females. In pairs with completely heterospecific *D. virilis* males (Group 2), a sharp increase in licking latency was observed. Furthermore, in some tests with completely heterospecific females (Group 4), the *D. americana* phenotype emerged, i.e., without any licking latency. Consequently, the increase in licking latency values did not reach a significant level. In pairs featuring males with a constant *D. virilis* genotype and females with a non-constant genotype (Group 3), a meaningful increase in licking latency is observed only in completely heterospecific pairs.

The presence of licking latency in pairs in which at least one partner has a complete or partial *D. virilis* genotype suggests the dominance of the *D. virilis* genotype for this trait. The increase in licking latency values to their maximum values in quartile estimates in entirely heterospecific pairs indicates an erratic and delayed execution of licking, and may even terminate the ritual at this stage or earlier.

The male singing duration is substantially longer in *D. americana* than in *D. virilis* males. Changes in chromosome composition in males courting females with a constant *D. virilis* genotype (Group 1) have no significant effect on the male singing duration. However, in experimental Group 3, alterations in chromosome composition in females courted by *D. virilis* males led to a significant drop in the male singing duration, reaching a minimum value in completely heterospecific pairs ♂ *D. virilis* + ♀ *D. americana*. In pairs with a constant *D. americana* genotype in males (Group 4), the male singing duration reaches a maximal value with *D. virilis* females and females from the backcross ♀ *D. americana* × ♂ F_1. It is likely that the *D. virilis* male detects a female of a different species and greatly decreases the courtship intensity, while the *D. americana* male persists in courting the female of a different species, raising the level of courtship intensity. Changes in the chromosome composition in a male paired with a female of the species *D. americana* (Group 2) results in a significant increase in the variability of the male singing duration.

This may explain the latency growth with an increase in the proportion of the *D. americana* genotype in males when paired with females with a constant *D. virilis* genotype (Group 1). In pairs with females with a constant *D. americana* genotype (Group 2), an increase in the proportion of the *D. virilis* genotype leads to a decrease in latency, which, in the case of F_2, closely approaches at its minimum the values of *D. virilis*, but without reaching these, increases again in the heterospecific variant to the values of *D. americana*. The influence of the male genotype on the male singing latency is much more pronounced than the influence of the female genotype.

On the contrary, the female singing duration is longer in *D. virilis* than in *D. americana*. A change in the chromosome composition of a male paired with a *D. virilis* female (Group 1) does not affect the duration of this element in any experiment, except for the completely heterospecific test variant, where a significant increase in duration is observed. Changing the chromosome composition of a female paired with a *D. virilis* male (Group 3) decreases the duration of female singing, except for the case where females were from the backcross ♀ *D. virilis* × ♂ F_1. The decrease is confirmed for pairs with F_1 females. In other cases, obtaining significant estimates is difficult due to the high variance of the trait.

An irregular growth in the duration of female singing was observed when the proportion of *D. virilis* chromosomes increased in pairs with a partner with a constant *D. americana* genotype (Groups 2 and 4), which depended on the chromosome composition of the second partner. The strongest confirmation of growth was obtained for pairs with a non-constant chromosome composition in females (Group 4).

The dependence of the female singing latency on the chromosome composition of the two species was observed only in pairs with a partner with a constant *D. virilis* genotype (Groups 1 and 3). Regardless of whether a male or a female act as the partner with a constant genotype, the female singing latency is always significantly greater for heterospecific pairs (*D. virilis* + *D. americana*) compared to conspecific pairs. The variability of this trait in pairs with hybrid partners depends on the chromosome composition and differs between males and females.

The "circling" element is characteristic of *D. virilis* males and appears at later stages of the ritual in conspecific pairs. In pairs with *D. virilis* females (Group 1), an increase in the proportion of *D. americana* chromosomes in males leads to a decrease in circling latency (backcross males) and then to a significant increase, up to the maximum latency (1801 sec), in F_1 males due to the absence of the "circling" element in most pairs. However, the circling duration shows no significant differences in all tests. In pairs with *D. virilis* males (Group 3), an increase in the proportion of *D. americana* chromosomes in females immediately leads to an increase in the median value of the circling duration, reaching maximum values, and a complete absence of the circling element in pairs with F_1 and *D. americana* females. The median value of the circling duration decreased sharply in pairs with hybrid females, but the high dispersion of values prevents obtaining a significant estimate of differences. *D. americana* males (Group 4) do not

exhibit circling in conspecific pairs but show it in pairs with backcrosses, F_2, and *D. virilis* females. Analyzing the median value of the circling duration in pairs with a *D. americana* female (Group 2), we observe extended circling only with males from backcrosses (♀ F_1 × ♂ *D. americana*). The dependence of the execution of the circling element on the sex of the partner with a constant genotype in the tested pairs indicates the effect of the sex chromosomes on the parameter variability of this element.

Table S1. Variability of the courtship elements in pairs ♀D. virilis + ♂D. virilis.

Traits	N	Mean	Median	Min	Max	Lower quartile	Upper quartile
Latency							
Following	30	1383.3	1801.0	0	1801	1801.0	1801.0
Tapping	30	0.0	0.0	0	1	0.0	0.0
Licking	30	5.7	2.0	0	80	1.0	4.0
Male singing	30	3.3	1.0	0	54	0.0	2.0
Circling	30	911.9	954.0	0	1801	2.0	1801.0
Copulation attempt	30	1383.1	1801.0	3	1801	1801.0	1801.0
Copulation	30	54.0	16.0	2	401	6.0	58.0
Female singing	30	0.9	1.0	0	4	0.0	1.0
Duration							
Following	30	2.3	0.0	0	45	0.0	0.0
Tapping	30	25.8	10.0	2	160	5.0	20.0
Licking	30	21.1	7.0	1	142	3.0	15.0
Male singing	30	15.5	7.0	1	94	3.0	15.0
Circling	30	1.9	1.0	0	17	0.0	3.0
Copulation attempt	30	5.9	0.0	0	75	0.0	0.0
Copulation	30	152.8	161.5	65	232	135.0	181.0
Female singing	30	13.4	6.5	1	139	3.0	14.0

N – the total number of the tested pairs; latency and duration are given in seconds

Table S2. Variability of the courtship elements in pairs ♀ D. virilis + ♂ D. americana.

Traits	N	Mean	Median	Min	Max	Lower quartile	Upper quartile
Latency							
Following	30	1122.2	1801.0	0	1801	91.0	1801.0
Tapping	30	62.4	0.0	0	1801	0.0	0.0
Licking	30	198.5	3.0	0	1801	0.0	17.0
Male singing	30	292.7	9.5	1	1801	3.0	36.0
Circling	30	956.0	1281.5	1	1801	59.0	1801.0
Copulation attempt	30	1209.9	1801.0	6	1801	30.0	1801.0
Copulation	30	1218.8	1801.0	4	1801	275.0	1801.0
Female singing	30	79.1	1.5	0	1801	1.0	7.0
Duration							
Following	30	4.2	0.0	0	42	0.0	2.0
Tapping	30	59.7	27.0	0	329	16.0	64.0
Licking	30	49.9	16.5	0	320	9.0	55.0
Male singing	30	18.4	10.0	0	109	2.0	12.0
Circling	30	8.1	1.0	0	108	0.0	5.0
Copulation attempt	30	3.5	0.0	0	41	0.0	1.0
Copulation	30	62.1	0.0	0	199	0.0	164.0
Female singing	30	40.7	14.0	0	435	5.0	30.0

N – the total number of the tested pairs; latency and duration are given in seconds

Table S3. Variability of the courtship elements in pairs ♀ *D. virilis* + ♂ F_1.

Traits	N	Mean	Median	Min	Max	Lower quartile	Upper quartile
Latency							
Following	30	1092.3	1801.0	0	1801	4.0	1801.0
Tapping	30	2.6	0.0	0	73	0.0	0.0
Licking	30	7.0	2.5	0	73	1.0	5.0
Male singing	30	164.7	7.5	0	1801	3.0	26.0
Circling	30	1371.6	1801.0	0	1801	494.0	1801.0
Copulation attempt	30	1249.1	1801.0	8	1801	66.0	1801.0
Copulation	30	477.7	43.0	4	1801	14.0	503.0
Female singing	30	27.2	3.0	0	590	0.0	7.0
Duration							
Following	30	3.7	0.0	0	48	0.0	4.0
Tapping	30	61.7	15.0	3	306	7.0	87.0
Licking	30	42.1	10.5	2	272	5.0	26.0
Male singing	30	14.5	4.0	0	122	3.0	10.0
Circling	30	1.2	0.0	0	7	0.0	2.0
Copulation attempt	30	4.8	0.0	0	38	0.0	1.0
Copulation	30	129.2	162.0	0	224	131.0	179.0
Female singing	30	13.3	6.0	1	118	3.0	14.0

N – the total number of the tested pairs; latency and duration are given in seconds

Table S4. : Variability of the courtship elements in pairs ♀ *D. virilis* + ♂ F_2.

Traits	N	Mean	Median	Min	Max	Lower quartile	Upper quartile
Latency							
Following	30	1468.8	1801.0	1	1801	1801.0	1801.0
Tapping	30	0.0	0.0	0	0	0.0	0.0
Licking	30	26.8	2.0	0	522	1.0	6.0
Male singing	30	159.3	3.5	0	1801	2.0	20.0
Circling	30	1035.3	1801.0	0	1801	3.0	1801.0
Copulation attempt	30	1514.2	1801.0	5	1801	1801.0	1801.0
Copulation	30	379.0	26.5	4	1801	9.0	264.0
Female singing	30	3.0	0.5	0	25	0.0	4.0
Duration							
Following	30	2.4	0.0	0	53	0.0	0.0
Tapping	30	82.2	20.5	4	896	8.0	45.0
Licking	30	63.2	10.0	2	665	4.0	30.0
Male singing	30	17.7	5.0	0	210	3.0	8.0
Circling	30	3.3	0.0	0	55	0.0	2.0
Copulation attempt	30	2.7	0.0	0	33	0.0	0.0
Copulation	30	147.6	157.5	0	293	102.0	207.0
Female singing	30	18.0	5.5	1	317	2.0	12.0

N – the total number of the tested pairs; latency and duration are given in seconds

Table S5 Variability of the courtship elements in pairs ♀ *D. virilis* + ♂ F_B (♀♀ *D. virilis* x ♂♂ F_1).

Traits	N	Mean	Median	Min	Max	Lower quartile	Upper quartile
Latency							
Following	30	1025.7	1801.0	0	1801	8.0	1801.0
Tapping	30	0.2	0.0	0	7	0.0	0.0
Licking	30	29.2	2.0	0	744	1.0	3.0
Male singing	30	5.3	3.0	0	43	1.0	4.0
Circling	30	845.0	288.5	0	1801	24.0	1801.0
Copulation attempt	30	1514.0	1801.0	6	1801	1801.0	1801.0
Copulation	30	218.9	36.5	4	1801	9.0	127.0
Female singing	30	5.1	1.0	0	73	0.0	4.0
Duration							
Following	30	4.9	0.0	0	33	0.0	7.0
Tapping	30	56.4	25.0	4	498	9.0	61.0
Licking	30	40.6	14.5	3	369	5.0	53.0
Male singing	30	18.9	11.5	1	84	4.0	21.0
Circling	30	2.8	2.5	0	16	0.0	3.0
Copulation attempt	30	5.1	0.0	0	46	0.0	0.0
Copulation	30	182.9	185.5	0	293	168.0	223.0
Female singing	30	14.1	7.0	1	103	3.0	14.0

N – the total number of the tested pairs; latency and duration are given in seconds

Table S6. Variability of the courtship elements in pairs ♀ *D. virilis* + ♂ F_B (♀♀ F_1 x ♂♂ *D. virilis*).

Traits	N	Mean	Median	Min	Max	Lower quartile	Upper quartile
Latency							
Following	30	1401.0	1801.0	0	1801	1801.0	1801.0
Tapping	30	0.4	0.0	0	8	0.0	0.0
Licking	30	34.9	2.5	0	799	2.0	5.0
Male singing	30	95.1	3.5	0	1801	2.0	6.0
Circling	30	701.1	59.0	0	1801	0.0	1801.0
Copulation attempt	30	1569.2	1801.0	14	1801	1801.0	1801.0
Copulation	30	174.5	9.0	4	1801	6.0	55.0
Female singing	30	28.6	1.5	0	801	0.0	3.0
Duration							
Following	30	1.4	0.0	0	14	0.0	0.0
Tapping	30	36.3	9.0	3	633	6.0	19.0
Licking	30	29.2	5.5	1	551	3.0	12.0
Male singing	30	10.1	4.0	0	80	2.0	11.0
Circling	30	2.6	2.0	0	31	0.0	2.0
Copulation attempt	30	2.5	0.0	0	62	0.0	0.0
Copulation	30	153.7	160.0	0	289	143.0	180.0
Female singing	30	8.4	4.0	1	50	1.0	9.0

N – the total number of the tested pairs; latency and duration are given in seconds

Table S7. Variability of the courtship elements in pairs ♀ *D. americana* + ♂ *D. virilis.*

Traits	N	Mean	Median	Min	Max	Lower quartile	Upper quartile
			Latency				
Following	30	857.6	368.0	0	1801	3.0	1801.0
Tapping	30	0.1	0.0	0	2	0.0	0.0
Licking	30	639.6	9.5	0	1801	1.0	1801.0
Male singing	30	771.4	82.5	0	1801	3.0	1801.0
Circling	30	1801.0	1801.0	1801	1801	1801.0	1801.0
Copulation attempt	30	1506.7	1801.0	3	1801	1801.0	1801.0
Copulation	30	1741.3	1801.0	10	1801	1801.0	1801.0
Female singing	30	96.4	2.5	0	1801	1.0	17.0
			Duration				
Following	30	1.3	1.0	0	6	0.0	2.0
Tapping	30	34.1	15.5	1	280	7.0	32.0
Licking	30	23.8	4.5	0	242	0.0	10.0
Male singing	30	7.2	1.5	0	44	0.0	9.0
Circling	30	0.0	0.0	0	0	0.0	0.0
Copulation attempt	30	1.1	0.0	0	15	0.0	0.0
Copulation	30	3.2	0.0	0	97	0.0	0.0
Female singing	30	7.0	3.0	0	26	1.0	15.0

N – the total number of the tested pairs; latency and duration are given in seconds

Table S8. Variability of the courtship elements in pairs ♀ *D. americana* + ♂ *D. americana.*

Traits	N	Mean	Median	Min	Max	Lower quartile	Upper quartile
Latency							
Following	30	1081.1	1801.0	0	1801	1.0	1801.0
Tapping	30	10.8	0.0	0	155	0.0	3.0
Licking	30	17.4	0.0	0	173	0.0	6.0
Male singing	30	34.0	10.5	2	174	5.0	38.0
Circling	30	1484.5	1801.0	0	1801	1801.0	1801.0
Copulation attempt	30	1590.5	1801.0	24	1801	1801.0	1801.0
Copulation	30	166.2	20.0	4	1801	8.0	89.0
Female singing	30	36.5	7.0	0	504	2.0	25.0
Duration							
Following	30	5.0	0.0	0	40	0.0	7.0
Tapping	30	26.3	11.0	4	194	7.0	27.0
Licking	30	25.6	10.5	4	183	7.0	27.0
Male singing	30	6.0	3.0	1	30	2.0	6.0
Circling	30	4.0	0.0	0	94	0.0	0.0
Copulation attempt	30	1.9	0.0	0	33	0.0	0.0
Copulation	30	164.7	148.0	0	380	134.0	181.0
Female singing	30	3.4	1.0	1	24	1.0	3.0

N – the total number of the tested pairs; latency and duration are given in seconds

Table S9. Variability of the courtship elements in pairs ♀ *D. americana* + ♂ F_1.

Traits	N	Mean	Median	Min	Max	Lower quartile	Upper quartile
Latency							
Following	30	1192.9	1801.0	0	1801	254.0	1801.0
Tapping	30	2.8	0.0	0	79	0.0	0.0
Licking	30	49.8	1.0	0	549	0.0	2.0
Male singing	30	118.0	7.5	0	1801	2.0	70.0
Circling	30	1566.4	1801.0	0	1801	1801.0	1801.0
Copulation attempt	30	1112.6	1801.0	5	1801	74.0	1801.0
Copulation	30	351.3	91.0	2	1801	11.0	504.0
Female singing	30	65.8	3.0	0	577	1.0	40.0
Duration							
Following	30	6.7	0.0	0	52	0.0	8.0
Tapping	30	53.5	28.5	2	258	11.0	64.0
Licking	30	42.1	19.5	2	241	9.0	48.0
Male singing	30	12.3	7.0	0	48	3.0	20.0
Circling	30	0.4	0.0	0	3	0.0	0.0
Copulation attempt	30	3.0	0.0	0	31	0.0	3.0
Copulation	30	149.0	152.0	0	299	135.0	169.0
Female singing	30	9.5	5.5	1	65	2.0	13.0

N – the total number of the tested pairs; latency and duration are given in seconds

Table S10. Variability of the courtship elements in pairs ♀ *D. americana* + ♂ F_2.

Traits	N	Mean	Median	Min	Max	Lower quartile	Upper quartile
Latency							
Following	30	1146.8	1801.0	0	1801	15.0	1801.0
Tapping	30	0.5	0.0	0	14	0.0	0.0
Licking	30	32.4	1.0	0	549	1.0	3.0
Male singing	30	127.8	5.5	0	1801	4.0	10.0
Circling	30	1047.4	1801.0	0	1801	14.0	1801.0
Copulation attempt	30	1563.4	1801.0	8	1801	1801.0	1801.0
Copulation	30	300.8	39.0	3	1801	8.0	208.0
Female singing	30	57.1	5.0	0	623	1.0	25.0
Duration							
Following	30	3.9	0.0	0	29	0.0	3.0
Tapping	30	41.0	19.5	2	330	7.0	47.0
Licking	30	33.9	11.5	3	293	5.0	43.0
Male singing	30	9.8	4.5	0	55	3.0	9.0
Circling	30	1.3	0.0	0	6	0.0	3.0
Copulation attempt	30	1.5	0.0	0	25	0.0	0.0
Copulation	30	120.9	127.5	0	219	107.0	143.0
Female singing	30	4.0	4.0	1	12	1.0	5.0

N – the total number of the tested pairs; latency and duration are given in seconds

Table S11. Variability of the courtship elements in pairs ♀ *D. americana* + ♂ F_B (♀♀ *D. americana* x ♂♂ F_1).

Traits	N	Mean	Median	Min	Max	Lower quartile	Upper quartile
			Latency				
Following	30	1213.0	1801.0	0	1801	44.0	1801.0
Tapping	30	2.2	0.0	0	66	0.0	0.0
Licking	30	34.8	1.0	0	775	1.0	2.0
Male singing	30	55.3	3.0	0	781	1.0	10.0
Circling	30	1131.5	1801.0	0	1801	79.0	1801.0
Copulation attempt	30	1508.7	1801.0	4	1801	1801.0	1801.0
Copulation	30	227.0	19.0	3	1801	5.0	129.0
Female singing	30	41.3	2.0	0	783	1.0	14.0
			Duration				
Following	30	5.4	0.0	0	44	0.0	4.0
Tapping	30	33.8	17.5	3	139	5.0	43.0
Licking	30	26.7	15.0	2	114	5.0	32.0
Male singing	30	7.9	4.0	1	39	2.0	9.0
Circling	30	1.8	0.0	0	22	0.0	2.0
Copulation attempt	30	1.2	0.0	0	19	0.0	0.0
Copulation	30	133.9	135.0	0	233	124.0	156.0
Female singing	30	5.6	3.0	1	36	1.0	7.0

N – the total number of the tested pairs; latency and duration are given in seconds

Table S12. Variability of the courtship elements in pairs ♀ *D. americana* + ♂ F_B (♀♀ F_1 x ♂♂ *D. americana*).

Traits	N	Mean	Median	Min	Max	Lower quartile	Upper quartile
Latency							
Following	30	814.5	165.0	0	1801	6.0	1801.0
Tapping	30	1.0	0.0	0	29	0.0	0.0
Licking	30	10.1	2.0	0	131	1.0	4.0
Male singing	30	171.6	12.0	0	1801	5.0	114.0
Circling	30	819.0	245.5	0	1801	1.0	1801.0
Copulation attempt	30	1365.9	1801.0	9	1801	1168.0	1801.0
Copulation	30	549.6	150.0	8	1801	27.0	1033.0
Female singing	30	35.4	3.0	0	561	1.0	9.0
Duration							
Following	30	10.9	2.5	0	105	0.0	11.0
Tapping	30	101.1	32.0	8	609	15.0	110.0
Licking	30	76.9	23.5	3	514	11.0	71.0
Male singing	30	14.9	10.5	0	76	4.0	14.0
Circling	30	6.8	2.0	0	46	0.0	7.0
Copulation attempt	30	1.4	0.0	0	21	0.0	1.0
Copulation	30	115.5	129.5	0	232	95.0	149.0
Female singing	30	10.6	6.0	1	43	3.0	16.0

N – the total number of the tested pairs; latency and duration are given in seconds

Table S13. Variability of the courtship elements in pairs ♀ F_1 + ♂ *D. americana*.

Traits	N	Mean	Median	Min	Max	Lower quartile	Upper quartile
Latency							
Following	30	1285.0	1801.0	0	1801	313.0	1801.0
Tapping	30	2.7	0.0	0	68	0.0	0.0
Licking	30	225.8	4.0	0	1801	1.0	63.0
Male singing	30	401.4	12.0	0	1801	6.0	261.0
Circling	30	1168.5	1801.0	0	1801	71.0	1801.0
Copulation attempt	30	1404.4	1801.0	2	1801	1801.0	1801.0
Copulation	30	864.3	349.5	6	1801	21.0	1801.0
Female singing	30	225.7	3.0	0	1801	2.0	64.0
Duration							
Following	30	2.4	0.0	0	19	0.0	4.0
Tapping	30	30.9	17.5	2	107	8.0	52.0
Licking	30	21.3	10.0	0	79	5.0	27.0
Male singing	30	5.5	3.0	0	31	1.0	5.0
Circling	30	3.4	0.0	0	29	0.0	3.0
Copulation attempt	30	5.5	0.0	0	61	0.0	0.0
Copulation	30	87.6	62.5	0	250	0.0	170.0
Female singing	30	12.7	5.0	0	107	1.0	9.0

N – the total number of the tested pairs; latency and duration are given in seconds

Table S14. Variability of the courtship elements in pairs ♀ F_1 + ♂ *D. virilis*.

Traits	N	Mean	Median	Min	Max	Lower quartile	Upper quartile
Latency							
Following	30	1082.5	1801.0	0	1801	1.0	1801.0
Tapping	30	1.2	0.0	0	23	0.0	0.0
Licking	30	9.9	2.0	0	110	1.0	7.0
Male singing	30	21.0	2.0	0	427	0.0	3.0
Circling	30	1445.5	1801.0	0	1801	1801.0	1801.0
Copulation attempt	30	1234.0	1801.0	2	1801	44.0	1801.0
Copulation	30	139.9	8.0	2	1801	5.0	125.0
Female singing	30	30.6	2.0	0	621	0.0	5.0
Duration							
Following	30	5.7	0.0	0	55	0.0	6.0
Tapping	30	21.1	6.5	2	262	4.0	20.0
Licking	30	16.6	3.5	1	238	3.0	16.0
Male singing	30	10.0	4.0	2	104	3.0	8.0
Circling	30	0.5	0.0	0	4	0.0	0.0
Copulation attempt	30	11.1	0.0	0	204	0.0	1.0
Copulation	30	131.8	134.0	0	189	125.0	148.0
Female singing	30	3.3	2.0	1	20	1.0	3.0

N – the total number of the tested pairs; latency and duration are given in seconds

Table S15. Variability of the courtship elements in pairs ♀ F_2 + ♂ *D. virilis.*

Traits	N	Mean	Median	Min	Max	Lower quartile	Upper quartile
Latency							
Following	30	1203.5	1801.0	0	1801	13.0	1801.0
Tapping	30	4.4	0.0	0	99	0.0	0.0
Licking	30	8.1	2.0	0	104	1.0	5.0
Male singing	30	6.3	2.0	0	103	1.0	3.0
Circling	30	1212.2	1801.0	0	1801	95.0	1801.0
Copulation attempt	30	1211.5	1801.0	2	1801	33.0	1801.0
Copulation	30	176.0	16.5	3	1801	7.0	140.0
Female singing	30	8.0	3.0	0	120	1.0	6.0
Duration							
Following	30	5.8	0.0	0	58	0.0	8.0
Tapping	30	20.6	9.5	3	169	5.0	16.0
Licking	30	12.9	5.0	1	86	3.0	12.0
Male singing	30	9.8	5.0	1	56	3.0	9.0
Circling	30	2.2	0.0	0	29	0.0	3.0
Copulation attempt	30	5.6	0.0	0	59	0.0	2.0
Copulation	30	124.7	137.5	0	184	112.0	151.0
Female singing	30	6.1	3.0	1	41	2.0	7.0

N – the total number of the tested pairs; latency and duration are given in seconds

Table S16. Variability of the courtship elements in pairs ♀ F_2 + ♂ *D. americana.*

Traits	N	Mean	Median	Min	Max	Lower quartile	Upper quartile
Latency							
Following	30	1040.5	1801.0	0	1801	7.0	1801.0
Tapping	30	0.1	0.0	0	2	0.0	0.0
Licking	30	67.8	3.0	0	719	1.0	5.0
Male singing	30	135.8	7.0	0	1801	4.0	18.0
Circling	30	766.3	133.0	0	1801	2.0	1801.0
Copulation attempt	30	1461.3	1801.0	6	1801	1801.0	1801.0
Copulation	30	377.1	25.0	5	1801	11.0	381.0
Female singing	30	20.9	3.0	0	199	1.0	13.0
Duration							
Following	30	6.2	0.0	0	62	0.0	4.0
Tapping	30	33.9	19.0	5	131	11.0	38.0
Licking	30	21.1	9.0	4	80	7.0	21.0
Male singing	30	7.6	4.0	0	29	3.0	11.0
Circling	30	4.8	2.5	0	40	0.0	7.0
Copulation attempt	30	7.5	0.0	0	189	0.0	0.0
Copulation	30	117.2	144.0	0	203	81.0	159.0
Female singing	30	11.8	4.0	1	119	2.0	10.0

N – the total number of the tested pairs; latency and duration are given in seconds

Table S17. Variability of the courtship elements in pairs ♀ F_B (♀♀*D. virilis* x ♂♂ F_1) + ♂ *D. virilis.*

Traits	N	Mean	Median	Min	Max	Lower quartile	Upper quartile
Latency							
Following	30	1684.2	1801.0	16	1801	1801.0	1801.0
Tapping	30	0.0	0.0	0	0	0.0	0.0
Licking	30	3.7	3.5	0	18	1.0	5.0
Male singing	30	10.6	2.5	0	127	1.0	5.0
Circling	30	1264.5	1801.0	0	1801	31.0	1801.0
Copulation attempt	30	1149.5	1801.0	7	1801	18.0	1801.0
Copulation	30	179.3	20.0	4	1801	9.0	130.0
Female singing	30	7.1	1.5	0	128	0.0	5.0
Duration							
Following	30	0.2	0.0	0	5	0.0	0.0
Tapping	30	62.7	14.0	4	579	9.0	43.0
Licking	30	48.8	12.5	2	500	4.0	33.0
Male singing	30	19.4	9.0	2	139	5.0	18.0
Circling	30	1.7	0.0	0	15	0.0	3.0
Copulation attempt	30	6.0	0.0	0	58	0.0	4.0
Copulation	30	170.7	179.5	0	285	145.0	217.0
Female singing	30	21.1	6.0	1	200	3.0	13.0

N – the total number of the tested pairs; latency and duration are given in seconds

Table S18. Variability of the courtship elements in pairs ♀ F_B (♀♀ *D. americana* x ♂♂ F_1) + ♂ *D. americana.*

Traits	N	Mean	Median	Min	Max	Lower quartile	Upper quartile
			Latency				
Following	30	724.0	126.5	0	1801	4.0	1801.0
Tapping	30	1.9	0.0	0	39	0.0	0.0
Licking	30	66.0	3.0	1	656	2.0	6.0
Male singing	30	143.2	11.5	2	1801	5.0	56.0
Circling	30	850.7	512.5	0	1801	84.0	1801.0
Copulation attempt	30	1002.6	1801.0	5	1801	19.0	1801.0
Copulation	30	563.7	246.0	5	1801	28.0	828.0
Female singing	30	53.3	5.0	0	622	1.0	24.0
			Duration				
Following	30	7.4	2.0	0	61	0.0	9.0
Tapping	30	62.7	38.0	5	248	15.0	91.0
Licking	30	38.1	22.0	4	157	11.0	49.0
Male singing	30	11.6	8.5	0	46	4.0	18.0
Circling	30	4.8	2.0	0	28	0.0	6.0
Copulation attempt	30	6.9	0.5	0	56	0.0	6.0
Copulation	30	112.8	124.0	0	237	87.0	156.0
Female singing	30	16.0	10.5	1	73	4.0	19.0

N – the total number of the tested pairs; latency and duration are given in seconds

Table S19. Variability of the courtship elements in pairs ♀ F_B (♀♀ F_1 x ♂♂ *D. virilis*) + ♂ *D. virilis.*

Traits	N	Mean	Median	Min	Max	Lower quartile	Upper quartile
Latency							
Following	30	1562.1	1801.0	2	1801	1801.0	1801.0
Tapping	30	1.3	0.0	0	39	0.0	0.0
Licking	30	2.1	1.0	0	10	0.0	3.0
Male singing	30	1.8	1.0	0	9	0.0	3.0
Circling	30	1097.1	1801.0	0	1801	1.0	1801.0
Copulation attempt	30	1445.0	1801.0	3	1801	1801.0	1801.0
Copulation	30	103.5	6.5	2	1801	4.0	32.0
Female singing	30	0.7	0.0	0	4	0.0	1.0
Duration							
Following	30	3.3	0.0	0	69	0.0	0.0
Tapping	30	29.0	6.5	2	218	4.0	21.0
Licking	30	21.7	3.5	2	147	3.0	18.0
Male singing	30	14.9	4.0	1	126	3.0	17.0
Circling	30	2.9	0.0	0	25	0.0	3.0
Copulation attempt	30	6.6	0.0	0	68	0.0	0.0
Copulation	30	147.5	149.0	0	209	136.0	168.0
Female singing	30	5.3	2.0	1	36	1.0	5.0

N – the total number of the tested pairs; latency and duration are given in seconds

Table S20. Variability of the courtship elements in pairs ♀ F_B (♀♀ F_1 x ♂♂ *D. americana*) + ♂ *D. americana.*

Traits	N	Mean	Median	Min	Max	Lower quartile	Upper quartile
Latency							
Following	30	1128.7	1801.0	0	1801	6.0	1801.0
Tapping	30	5.7	0.0	0	125	0.0	0.0
Licking	30	71.0	4.0	1	826	1.0	33.0
Male singing	30	192.3	17.5	1	1638	5.0	185.0
Circling	30	906.3	904.5	0	1801	1.0	1801.0
Copulation attempt	30	1517.1	1801.0	7	1801	1801.0	1801.0
Copulation	30	533.1	51.5	5	1801	12.0	877.0
Female singing	30	23.7	2.5	0	271	1.0	9.0
Duration							
Following	30	5.2	0.0	0	107	0.0	3.0
Tapping	30	36.0	18.5	5	228	12.0	47.0
Licking	30	15.7	10.0	3	153	6.0	16.0
Male singing	30	6.6	4.0	1	55	3.0	7.0
Circling	30	4.3	2.0	0	34	0.0	5.0
Copulation attempt	30	5.1	0.0	0	73	0.0	0.0
Copulation	30	95.5	114.0	0	193	60.0	143.0
Female singing	30	9.5	6.0	1	62	3.0	11.0

N – the total number of the tested pairs; latency and duration are given in seconds

Table S21. The effect of the male genotype on variability of the courtship elements in pairs with *D. virilis* female.

Traits	K-W H, p
Latency	
Following	H (5, N= 180) =7.181447 p =.2075
Tapping	H (5, N= 180) =3.825658 p =.5748
Licking	H (5, N= 180) =1.763032 p =.8809
Male singing	**H (5, N= 180) =41.98996 p =.0000**
Circling	H (5, N= 180) =14.71774 p =.0116
Copulation attempt	H (5, N= 180) =6.308060 p =.2774
Copulation	**H (5, N= 180) =42.04041 p =.0000**
Female singing	H (5, N= 180) =15.32026 p =.0091
Duration	
Following	H (5, N= 180) =8.592653 p =.1265
Tapping	H (5, N= 180) =17.83552 p =.0032
Licking	H (5, N= 180) =14.41868 p =.0132
Male singing	H (5, N= 180) =7.040775 p =.2176
Circling	H (5, N= 180) =7.831924 p =.1657
Copulation attempt	H (5, N= 180) =5.165331 p =.3960
Copulation	**H (5, N= 180) =30.10578 p =.0000**
Female singing	H (5, N= 180) =12.21365 p =.0320

Table S22. The effect of the male genotype on variability of the courtship elements in pairs with *D. americana* female.

Traits	K-W H, p
Latency	
Following	H (5, N= 180) =5.778818 p =.3283
Tapping	H (5, N= 180) =11.86424 p =.0367
Licking	**H (5, N= 180) =20.61970 p =.0010**
Male singing	H (5, N= 180) =19.00716 p =.0019
Circling	**H (5, N= 180) =33.49921 p =.0000**
Copulation attempt	H (5, N= 180) =9.372011 p =.0951
Copulation	**H (5, N= 180) =64.91437 p =.0000**
Female singing	H (5, N= 180) =3.861572 p =.5695
Duration	
Following	H (5, N= 180) =4.885943 p =.4300
Tapping	H (5, N= 180) =15.76250 p =.0076
Licking	**H (5, N= 180) =24.66141 p =.0002**
Male singing	H (5, N= 180) =20.37544 p =.0011
Circling	**H (5, N= 180) =33.51017 p =.0000**
Copulation attempt	H (5, N= 180) =8.418367 p =.1346
Copulation	**H (5, N= 180) =72.14546 p =.0000**
Female singing	**H (5, N= 180) =21.50413 p =.0007**

Table S23. The effect of the female genotype on variability of the courtship elements in pairs with *D. virilis* male.

Traits	K-W H, p
Latency	
Following	**H (5, N= 180) =23.42355 p =.0003**
Tapping	H (5, N= 180) =6.411942 p =.2682
Licking	H (5, N= 180) =15.34918 p =.0090
Male singing	**H (5, N= 180) =39.89650 p =.0000**
Circling	**H (5, N= 180) =21.05608 p =.0008**
Copulation attempt	H (5, N= 180) =4.612226 p =.4650
Copulation	**H (5, N= 180) =70.37235 p =.0000**
Female singing	**H (5, N= 180) =30.80197 p =.0000**
Duration	
Following	H (5, N= 180) =19.27938 p =.0017
Tapping	H (5, N= 180) =13.71291 p =.0175
Licking	H (5, N= 180) =10.13644 p =.0715
Male singing	**H (5, N= 180) =20.98000 p =.0008**
Circling	**H (5, N= 180) =20.91158 p =.0008**
Copulation attempt	H (5, N= 180) =4.614664 p =.4647
Copulation	**H (5, N= 180) =87.65231 p =.0000**
Female singing	**H (5, N= 180) =22.68250 p =.0004**

Table S24. The effect of the female genotype on variability of the courtship elements in pairs with *D. americana* male.

Traits	K-W H, p
	Latency
Following	H (5, N= 180) =5.710857 p =.3354
Tapping	H (5, N= 180) =9.159639 p =.1029
Licking	H (5, N= 180) =14.48424 p =.0128
Male singing	H (5, N= 180) =3.123600 p =.6809
Circling	H (5, N= 180) =14.39751 p =.0133
Copulation attempt	H (5, N= 180) =11.83200 p =.0372
Copulation	**H (5, N= 180) =34.43023 p =.0000**
Female singing	H (5, N= 180) =7.658934 p =.1761
	Duration
Following	H (5, N= 180) =6.641454 p =.2487
Tapping	H (5, N= 180) =15.23183 p =.0094
Licking	H (5, N= 180) =10.62658 p =.0593
Male singing	**H (5, N= 180) =20.34657 p =.0011**
Circling	H (5, N= 180) =12.04429 p =.0342
Copulation attempt	H (5, N= 180) =11.41955 p =.0437
Copulation	H (5, N= 180) =19.39956 p =.0016
Female singing	**H (5, N= 180) =34.91850 p =.0000**

Table S25. Results of the Mann-Whitney U Test

	Rank Sum – No	Rank Sum – Yes	U	Z	p-level	Z – adj.	p-level	Valid N - No	Valid N - Yes	2*1 sided - exact p
**Following**	858.0	972.0	393.0	-0.84	0.399	-1.040	0.298	30	30	0.406
**Tapping**	884.0	946.0	419.0	-0.46	0.647	-0.46	0.646	30	30	0.654
**Licking**	876.0	954.0	411.0	-0.58	0.564	-0.58	0.564	30	30	0.572
**Male singing**	832.5	997.5	367.5	-1.22	0.223	-1.23	0.219	30	30	0.224
**Circling**	908.5	921.5	443.5	-0.10	0.923	-0.11	0.914	30	30	0.924
**Copulation**	954.0	876.0	411.0	0.58	0.564	0.58	0.564	30	30	0.572
**Female singing**	1036.0	794.0	329.0	1.79	0.074	1.81	0.071	30	30	0.075

Table S26. Results of the Wald-Wolfowitz Runs Test

	Valid N - No	Valid N - Yes	Mean - No	Mean - Yes	Z	p-level	Z adjstd	p-level	No. of Runs	No. of ties
**Following**	30	30	4.03	3.87	0.00	1.00	-0.13	0.90	31	24
**Tapping**	30	30	28.70	40.97	-0.26	0.79	0.13	0.90	30	7
**Licking**	30	30	18.90	33.93	1.30	0.19	1.17	0.24	36	17
**Male singing**	30	30	5.83	9.80	-0.26	0.79	0.13	0.90	30	20
**Circling**	30	30	1.50	1.33	-0.26	0.79	0.13	0.90	30	26
**Copulation**	30	30	119.83	120.87	-1.04	0.10	0.91	0.36	27	10
**Female singing**	30	30	10.70	3.97	-0.26	0.79	0.13	0.90	30	20

Table S27. Evaluation of model fitting for data sets pooled in accordance with male or female homology (groups 1-4).

Traits	Fixed mate genotype	Model, method	RMR	GFI	AGFI	PGFI
Latency	♀ *D. virilis (group 1)*	14, ULS	**0.036**	**0.983**	**0.97**	**0.572**
		14, SLS	0.043	0.862	0.763	0.502
		16, ULS	**0.032**	**0.986**	**0.975**	**0.538**
		16, SLS	0.04	0.952	0.912	0.519
	♀ *D. americana (group 2)*	20, ULS	**0.024**	**0.994**	**0.987**	**0.47**
		20, SLS	0.032	0.968	0.933	0.458
Duration	♀ *D. virilis (group 1)*	23, ULS	**0.007**	**0.998**	**0.992**	**0.31**
		23, SLS	0.008	0.997	0.99	0.31
	♀ *D. americana (group 2)*	23, ULS	0.008	0.997	0.991	0.288
		23, SLS	0.008	0.995	0.982	0.287
Latency	♂ *D. virilis (group 3)*	23, ULS	**0.018**	**0.997**	**0.99**	**0.288**
		23, SLS	0.026	0.985	0.949	0.285
		25, ULS	**0.014**	**0.998**	**0.994**	**0.266**
		25, SLS	0.018	0.989	0.96	0.264
	♂ *D. americana (group 4)*	19, ULS	**0.025**	**0.994**	**0.986**	**0.398**
		19, SLS	0.029	0.969	0.923	0.388
		26, ULS	**0.022**	**0.997**	**0.988**	0.244
		26, SLS	0.03	0.993	0.973	0.243
Duration	♂ *D. virilis (group 3)*	27, ULS	**0.007**	**0.997**	**0.986**	0.222
		27, SLS	0.007	0.992	0.963	0.22
		32, ULS	**0.002**	**1**	**0.999**	**0.111**
		32, SLS	0.002	1	0.999	0.111
		sat.*, ULS	0.002	1	0.998	0.044
		sat.*, SLS	0.002	1	0.998	0.044
	♂ *D. americana (group 4)*	26, ULS	0.003	1	0.998	0.2
		26, SLS	0.003	0.999	0.995	0.2

sat.* (saturated) – as a saturated model characterizing by good match with the observed variability of duration traits of the courtship elements in pairs pooled by *D. virilis* males, a base model was used.
